# Atlas‐Guided CD55‐Enriched Neutrophil Extracellular Vesicles for Complement–Macrophage Modulation in Diabetic Wounds

**DOI:** 10.1002/advs.78320

**Published:** 2026-10-15

**Authors:** Tairong Cui, Jieying Li, Jibing He, Jia Wang, Yun Qian, Yilin Jin, Liuqing Yang, Zaijin Tao, Ziyun Li, Zhengsheng Chen, Wenjing Wang, Xin Ma, Jinglei Wu

**Affiliations:** ^1^ Department of Orthopedic Surgery Shanghai Sixth People's Hospital Affiliated to Shanghai Jiao Tong University School of Medicine Shanghai People's Republic of China; ^2^ Shanghai Engineering Research Center of Nano‐Biomaterials and Regenerative Medicine College of Biological Science and Medical Engineering Donghua University Shanghai People's Republic of China; ^3^ Fudan University School of Nursing Shanghai People's Republic of China; ^4^ Department of Clinical Laboratory Shanghai Sixth People's Hospital Affiliated to Shanghai Jiao Tong University School of Medicine Shanghai People's Republic of China

**Keywords:** atlas‐guided immunoengineering, complement regulation, diabetic foot ulcer, localized delivery, macrophage modulation, multi‐omics, single‐cell transcriptomics

## Abstract

Diabetic wounds are characterized by persistent inflammation and impaired immune resolution, yet the therapeutic translation of single‐cell atlases into rational immunomodulatory strategies remains limited. Here, integration of diabetic‐wound single‐cell transcriptomic datasets identifies a neutrophil subpopulation enriched in cluster of differentiation 55 (CD55) with complement‐regulatory and reparative features. Guided by this atlas‐derived signature, activated neutrophil‐derived extracellular vesicles (aNEVs) enriched in CD55 are generated under high‐glucose and lipopolysaccharide stimulation and incorporated into a microbial transglutaminase‐crosslinked gelatin hydrogel for localized delivery and protease‐sensitive matrix remodeling. The resulting gelatin/aNEV composite hydrogel (Gel/aNEV) attenuates complement‐associated inflammatory signaling, C3a production, oxidative stress, and pro‐inflammatory cytokine expression, while promoting reparative macrophage reprogramming and endothelial migration. In diabetic wounds, a single application of Gel/aNEV accelerates wound closure and enhances angiogenesis, collagen remodeling, and tissue regeneration. Integrated transcriptomic–proteomic analyses further reveal coordinated changes in complement, inflammatory, redox, extracellular‐matrix, and regenerative programs, supporting a multicomponent therapeutic model. Collectively, this study establishes an atlas‐to‐therapy framework that uses disease‐associated neutrophil states to guide extracellular‐vesicle engineering, integrating complement regulation, immune reprogramming, and biomaterial‐assisted delivery for diabetic wound repair.

## Graphical abstract

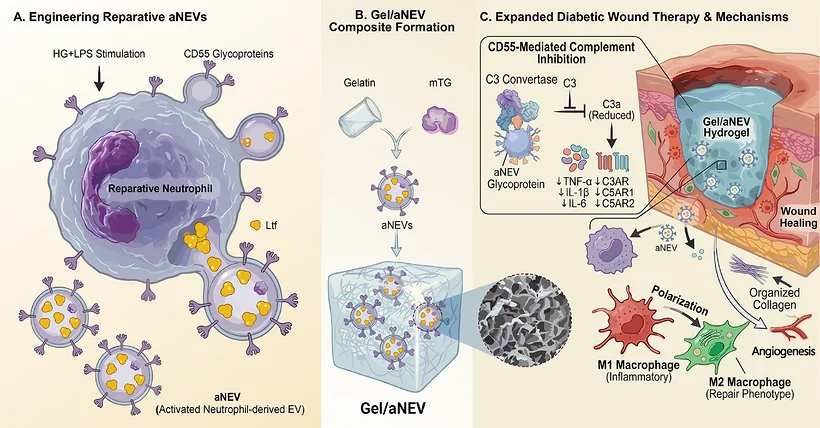

**Abbreviations**
AGE: advanced glycation end product
aNEV: activated neutrophil‐derived extracellular vesicle
ANOVA: analysis of variance
BCA: bicinchoninic acid
C3aR: C3a receptor
C5aR1: C5a receptor 1
C5aR2: C5a receptor 2
CCK‐8: cell counting kit‐8
CD55: cluster of differentiation 55 (decay‐accelerating factor)
cDNA: complementary DNA
CLON‐G: neutrophil survival cocktail comprising Q‐VD‐Oph, DFO, Hsp70, NAC, Nec‐1s, and G‐CSF
CR3: complement receptor 3
DAPI: 4′,6‐Diamidino‐2‐phenylindole
DEG: differentially expressed gene
DEP: differentially expressed protein
DFO: deferoxamine
DFU: diabetic foot ulcer
DM‐SWF: diabetic microenvironment–mimetic simulated wound fluid
DPN: diabetic peripheral neuropathy
ECM: extracellular matrix
ELISA: enzyme‐linked immunosorbent assay
EV: extracellular vesicle
EWR: equilibrium water absorption ratio
FBS: fetal bovine serum
FDR: false discovery rate
FITC: fluorescein isothiocyanate
G‐CSF: granulocyte colony‐stimulating factor
Gel/aNEV: gelatin/activated neutrophil‐derived extracellular vesicle composite hydrogel
GO: gene Ontology
GPI: glycosylphosphatidylinositol
GSEA: gene set enrichment analysis
GSVA: gene set variation analysis
H&E: hematoxylin and eosin
HBSS: Hanks’ balanced salt solution
HEK293: human embryonic kidney 293
HG: high glucose
Hsp70: heat shock protein 70
HUVEC: human umbilical vein endothelial cell
IF: immunofluorescence
IHC: immunohistochemistry
IL: interleukin
iPSC: induced pluripotent stem cell
KEGG: Kyoto Encyclopedia of Genes And Genomes
LAMP1: lysosome‐associated membrane protein 1
LAND‐V: large aging‐neutrophil‐derived vesicle
LC‐MS/MS: liquid chromatography–tandem mass spectrometry
LPS: lipopolysaccharide
MMP: matrix metalloproteinase
mTG: microbial transglutaminase
NAC: N‐acetylcysteine
Nec‐1s: Necrostatin‐1s
NET: neutrophil extracellular trap
NEV: neutrophil‐derived extracellular vesicle
NF‐κB: nuclear factor‐kappa B
NTA: nanoparticle tracking analysis
OD: optical density
PBS: phosphate‐buffered saline
POD: postoperative day
PPI: protein–protein interaction
qPCR: quantitative polymerase chain reaction
RAGE: receptor for advanced glycation end products
RNA‐seq: RNA sequencing
ROS: reactive oxygen species
RT‐qPCR: reverse transcription quantitative polymerase chain reaction
scRNA‐seq: single‐cell RNA sequencing
SD: standard deviation
SEM: scanning electron microscopy
SPF: specific pathogen‐free
STZ: streptozotocin
T2DM: type 2 diabetes mellitus
TEM: transmission electron microscopy
TGF: transforming growth factor
TNF: tumor necrosis factor
UMAP: uniform manifold approximation and projection
α‐SMA: alpha‐smooth muscle actin
ΔΔCt: delta‐delta cycle threshold

## 1 Introduction

Chronic wounds, exemplified by diabetic foot ulcers (DFUs), represent a state of failed immune resolution in which persistent inflammatory amplification overwhelms endogenous reparative programs [1, 2]. Single‐cell transcriptomics has substantially advanced the definition of cellular heterogeneity in chronic wounds, yet translating disease‐associated cell states into actionable therapeutic designs remains challenging. Bridging this “atlas‐to‐therapy” gap requires strategies that move beyond descriptive cell mapping toward the identification of molecular programs that can be reproduced in scalable, cell‐free therapeutic systems [3, 4].

Neutrophils are central to this unresolved inflammatory state but also exhibit substantial functional plasticity. In the diabetic microenvironment, persistent hyperglycemia, advanced glycation end products (AGEs), and oxidative stress prolong neutrophil activation [5, 6, 7], promoting excessive neutrophil extracellular trap formation and reactive oxygen species (ROS) production [8, 9]. These events reinforce macrophage inflammatory activation and impair the transition toward tissue‐reparative states, thereby compromising angiogenesis and extracellular‐matrix restoration [4, 10]. Importantly, neutrophils are not uniformly pathogenic. Context‐dependent neutrophil states can acquire regulatory and reparative functions [11], and neutrophil‐derived extracellular vesicles (NEVs) provide a cell‐free route for transferring bioactive signals, including miR‐223, lactoferrin (Ltf), annexin A1, lipoxin A4, and itaconate [12]. Senescent neutrophils have also been reported to release large vesicles positive for cluster of differentiation 55 (CD55) (LAND‐Vs; >1 µm) capable of attenuating complement activation through membrane‐associated CD55 [13]. However, LAND‐V production depends on aging neutrophils, and their large size may restrict tissue distribution, whereas freely administered small NEVs face rapid clearance and limited wound retention [14].

Extracellular vesicle (EV) therapies from other cellular sources further support the potential of cell‐free wound immunomodulation. Mesenchymal stromal cell‐derived EVs exert broad anti‐inflammatory and regenerative effects [15]. Platelet‐derived EVs have likewise demonstrated pro‐angiogenic, pro‐migratory, and immunomodulatory effects in diabetic wound models when incorporated into hydrogel‐based delivery systems [16]. More recently, platelet‐derived EVs have been incorporated into gelatin‐based hydrogels for wound‐care applications, further highlighting their translational potential [17]. Rather than seeking a universally superior EV source, these studies highlight that therapeutic function is strongly shaped by vesicle origin and molecular composition. In this context, neutrophil‐derived vesicles offer a distinct opportunity to couple vesicular immunoregulatory cargo with surface complement regulation. We therefore adopted a “waste‐to‐treasure” immunoengineering concept in which a disease‐associated neutrophil program is decoded and repurposed as a therapeutic blueprint rather than simply suppressed. Guided by single‐cell identification of a CD55‐enriched C2 neutrophil state, high‐glucose plus lipopolysaccharide (HG+LPS) conditioning was used to generate activated neutrophil‐derived extracellular vesicles (aNEVs) enriched in complement‐regulatory and immunomodulatory features. This design allows membrane‐associated CD55 and vesicular cargo, including Ltf, to be considered as complementary components of a multicomponent immunoregulatory system rather than attributing therapeutic activity to a single vesicular constituent.

Effective local delivery remains a major barrier to EV‐based wound therapy because freely administered vesicles are vulnerable to enzymatic degradation, washout, and insufficient retention within exudative wounds [17, 18]. To address this limitation, we incorporated aNEVs into a microbial transglutaminase‐crosslinked gelatin hydrogel (mTG‐Gel). mTG‐mediated crosslinking occurs under mild physiological conditions, avoiding harsh chemical processing that may compromise sensitive vesicular components [19, 20], while the intrinsic proteolytic susceptibility of gelatin permits progressive matrix remodeling in protease‐rich wound environments [21, 22, 23]. Accordingly, the Gel/aNEV platform was designed to integrate atlas‐guided neutrophil‐state engineering, local EV retention, complement regulation, and macrophage immunomodulation within a single cell‐free system. In the present study, we further examine the correspondence between HG+LPS‐conditioned neutrophils and the atlas‐derived C2 program, characterize aNEV incorporation and release from the hydrogel under wound‐relevant conditions, and investigate the coordinated contribution of complement‐associated and cargo‐associated signaling to diabetic wound repair.

## 2 Results and Discussion

### 2.1 Single‐Cell Transcriptomics Identifies a CD55‐Enriched Neutrophil Subcluster as a Therapeutic Target for Diabetic Wound Healing

Human diabetic foot ulcer (DFU) specimens exhibited prominent inflammatory infiltration and disorganized tissue architecture (Figure 2I). Hematoxylin and eosin (H&E) staining of clinical amputation specimens revealed dense infiltration of inflammatory cells with enlarged, hyperchromatic nuclei throughout the ulcer bed. This infiltrate, predominantly composed of neutrophils and monocytes/macrophages, was notably concentrated at the interface between necrotic and viable tissue, forming a distinct “inflammatory front.” Masson's trichrome staining further demonstrated highly disorganized dermal collagen fibers in DFU tissues. The normal dense, bundled architecture was degraded into fragmented or wavy structures, indicating impaired matrix remodeling (Figure 2I). Notably, significant heterogeneity was observed among human samples. Ulcers of the same Wagner grade presented vastly different inflammatory gradients and fibrosis levels across individuals. This variability may be associated with factors such as glycemic fluctuations, AGE burden, infection profiles, and intrinsic differences in individual immune backgrounds, posing a challenge for subsequent precision interventions [24].

To characterize the cellular heterogeneity of DFU inflammation and identify candidate targets for therapeutic engineering, publicly available human DFU scRNA‐seq datasets (GSM7908977 and GSM7908978), a mouse diabetic wound dataset from db/db mice on a C57BLKS/J background (GSM8013669), and normal control data (GSM7025081) were analyzed (Figures 1 and 2). The human and murine datasets were used to examine shared inflammatory features, with subsequent murine analyses resolving neutrophil subpopulation heterogeneity.

**FIGURE 1:**
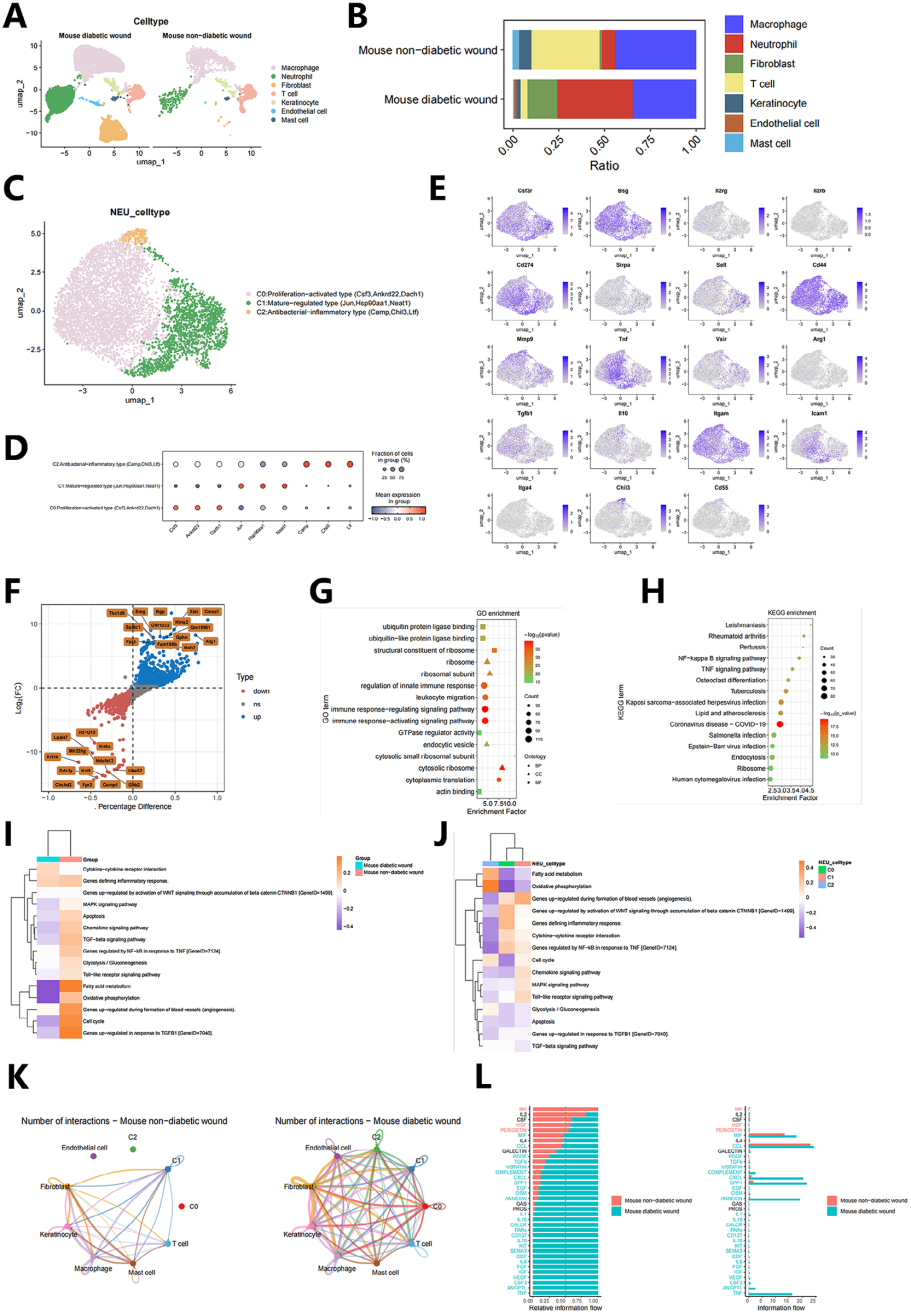
Single‐cell transcriptomic analysis of mouse diabetic wound samples from public databases. (A) Uniform Manifold Approximation and Projection (UMAP) visualization of cell clustering in normal control and diabetic wound samples. (B) Proportional composition of cellular components across experimental conditions. (C) and (D) Subclustering of neutrophil populations and visualization of highly variable genes. (E) Heatmap of gene expression clustering across neutrophil subpopulations. (F) Differential gene expression analysis of neutrophils between diabetic and control conditions. (G, H) Gene Ontology (GO) and Kyoto Encyclopedia of Genes and Genomes (KEGG) enrichment analyses of differentially expressed genes. (I, J) Gene Set Variation Analysis (GSVA) of neutrophil subpopulations. (K, L) Intercellular communication analysis between neutrophil subpopulations and other cell types.

**FIGURE 2:**
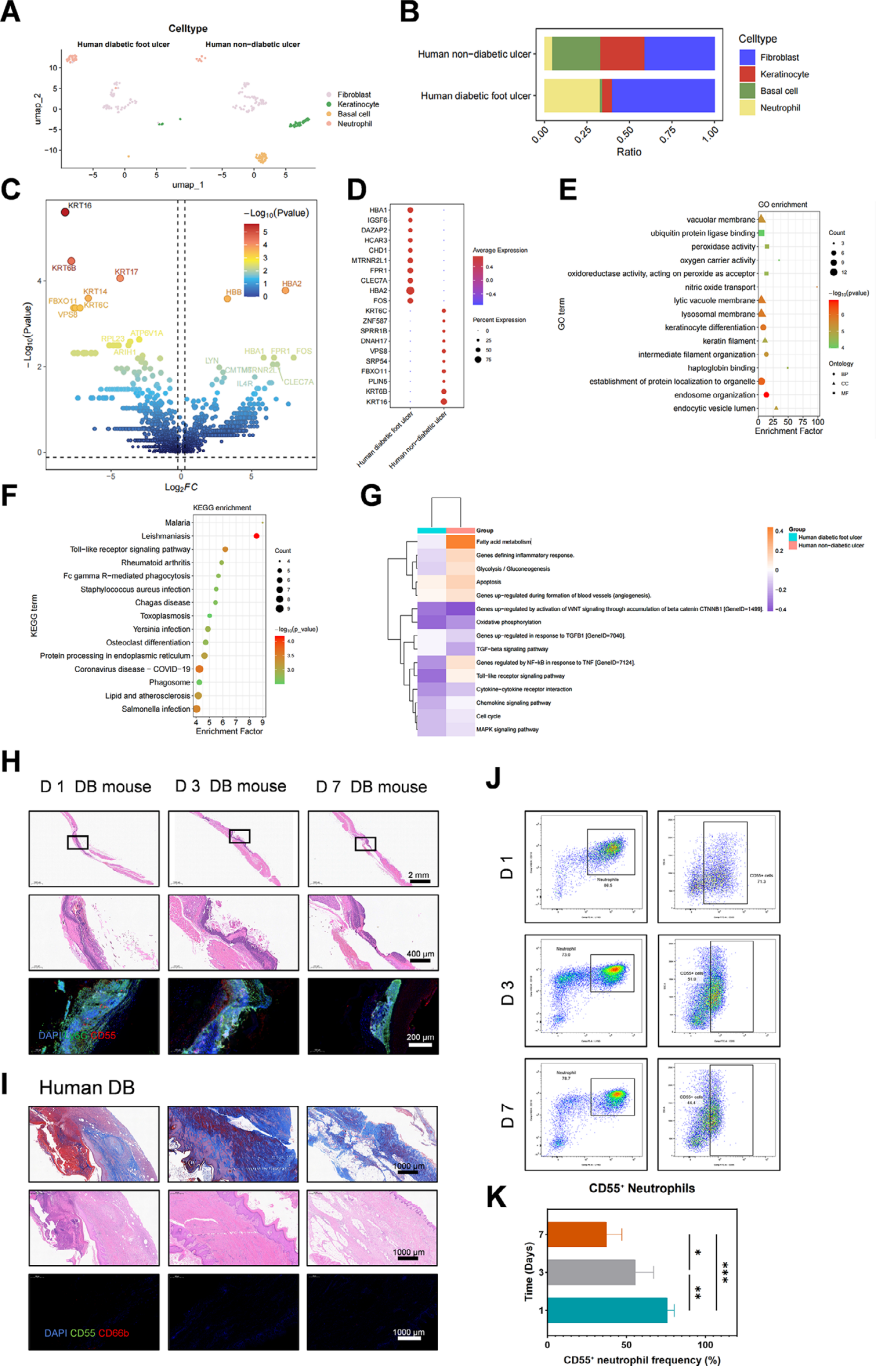
Single‐cell transcriptomic profiling of human diabetic and non‐diabetic wound samples and phenotypic validation of CD55^+^ neutrophils in murine and clinical models. (A–G) Single‐cell transcriptomic analysis of human diabetic foot ulcer and non‐diabetic wound samples from public databases: (A) Uniform Manifold Approximation and Projection (UMAP) visualization of cell clusters; (B) quantitative comparison of cell type proportions; (C) volcano plot depicting differentially expressed genes (DEGs); (D) dot plot of top 20 DEGs ranked by statistical significance; (E) Gene Ontology (GO) enrichment analysis of DEGs; (F) Kyoto Encyclopedia of Genes and Genomes (KEGG) pathway enrichment analysis; (G) Heatmap summarizing enrichment patterns of selected gene sets related to inflammation, metabolism, and tissue repair. (H) Representative H&E staining and immunofluorescence staining for Ly6G^+^CD55^+^ neutrophils in diabetic mouse wounds at days 1, 3, and 7 post‐injury. (I) Masson's trichrome staining, H&E staining, and immunofluorescence staining for CD66b^+^CD55^+^ neutrophils in human diabetic foot ulcer tissues. (J) Flow cytometric quantification of CD55^+^ neutrophils in diabetic mouse wound tissues. (K) Quantitative analysis of CD55^+^ neutrophil frequency among total neutrophils in diabetic mouse wound tissues. Data are mean ± SD (n = 5). ^*^ *p* < 0.05, ^**^ *p* < 0.01, ^***^ *p* < 0.001.

Following quality control and Harmony‐based integration, 4 major cell populations were identified in human samples and 7 in murine wounds. Diabetic samples showed a higher relative proportion of neutrophils in both the human and murine datasets, together with changes in the composition of other cell populations (Figures 1A,B and 2A,B). Differential expression and pathway enrichment analyses further supported enhanced inflammatory and stress‐associated signatures in diabetic wounds relative to controls (Figures 1F–H and 2C–G).

Given the limited number of neutrophils recovered from human samples, subsequent analyses focused on murine datasets to resolve neutrophil heterogeneity at higher resolution. Unsupervised clustering identified three transcriptionally distinct neutrophil subpopulations, designated as C0, C1, and C2 (Figure 1C,D). C0 exhibited a proliferative‐activated profile marked by *Csf3*, *Ankrd22*, and *Dach1* expression, whereas C1 displayed features of mature regulatory neutrophils characterized by *Jun*, *Hsp90aa1*, and *Neat1*. In contrast, the C2 subset was enriched for antimicrobial and inflammation‐associated genes, including *Camp*, *Chil3*, and *Ltf*. Although comprising only 6%–8% of total neutrophils, C2 displayed a unique transcriptional program suggestive of endogenous protective potential within the diabetic microenvironment.

Notably, Cd55 expression was preferentially enriched within the C2 subset (Figure 1E). Because Camp, Chil3, and Ltf encode intracellular or secreted proteins unsuitable for viable‐cell isolation [13, 16, 25, 26], CD55 was selected as a surrogate surface marker for downstream functional validation. CD55, also known as decay‐accelerating factor, is a glycosylphosphatidylinositol‐anchored complement regulator that restricts C3 and C5 convertase activity and protects host tissues from excessive complement activation [27]. The coexistence of anti‐inflammatory complement‐regulatory features and reparative transcriptional signatures identified C2 neutrophils as a promising template for therapeutic engineering.

Pseudotime trajectory inference by Monocle 2 reconstructed neutrophil differentiation dynamics, revealing that C2 neutrophils predominantly occupied terminal differentiation states with distinct gene expression kinetics along the pseudotime axis (Figure S1).

Intercellular communication analyses further suggested that C2 underwent marked phenotypic remodeling in diabetic wounds. Compared with the relatively quiescent state observed under non‐diabetic conditions, diabetic C2 neutrophils displayed enhanced interactions with endothelial cells, fibroblasts, macrophages, mast cells, and T cells, accompanied by increased signaling activity involving pathways associated with tissue remodeling and immune regulation (Figures 1K,L and S2). These findings support the notion that the C2 subset occupies a strategic position at the interface between inflammatory amplification and tissue adaptation during diabetic wound repair.

Based on the CD55 expression distribution identified in scRNA‐seq analysis, we subsequently examined CD55 protein expression in tissue specimens. In normal mouse skin, H&E staining revealed intact epidermal architecture with minimal inflammatory cell infiltration during early wound healing, while immunofluorescence showed sparse localization of Ly6G^+^CD55^+^ neutrophils restricted to the superficial wound edge, contrasting sharply with the dense, persistent CD55^+^ neutrophil accumulation observed in diabetic wounds (Figure S3). Immunofluorescence staining revealed substantial inter‐sample heterogeneity in CD55 expression levels across human tissues, with generally weak signal intensity. In contrast, mouse models exhibited clear time‐dependent expression patterns (Figure 2H,I).

Flow cytometric analysis revealed temporal changes in the frequency of CD55^+^ neutrophils in diabetic wound tissues. Among the examined time points, the proportion of CD55^+^ cells within the neutrophil population was highest on day 1 after injury (76.0% ± 4.3%) and declined to 55.7% ± 11.5% on day 3 and 37.4% ± 9.7% on day 7 (Figure 2J,K).

The declining frequency of CD55^+^ neutrophils over the first week of diabetic wound healing identifies a temporal feature of the endogenous CD55‐associated response. Together with the C2 transcriptional signature, this observation motivated the development of CD55‐enriched aNEVs as a local immunomodulatory strategy for diabetic wound repair.

### 2.2 HG+LPS Stimulation Partially Recapitulates the Atlas‐Derived C2 Program and Generates CD55‐Enriched aNEVs

Bone marrow‐derived neutrophils were isolated from 8‐week‐old male C57BL/6 mice by Percoll density gradient centrifugation, with purity exceeding 90% confirmed by flow cytometric detection of Ly6G surface expression (Figure S4A,C). Combined HG+LPS stimulation for 24 h induced the highest CD55 expression on neutrophils compared with single‐factor or unstimulated controls, establishing the ex vivo conditioning strategy subsequently used for aNEV production (Figure S4B,D). To determine whether this phenotype was molecularly linked to the atlas‐defined C2 state rather than representing nonspecific neutrophil activation, we performed targeted RT‐qPCR using a gene panel selected directly from the single‐cell‐derived neutrophil signatures. The panel comprised the C2‐associated genes *Cd55, Ltf, Chil3*, and *Camp*, together with *Csf3* and *Jun* as representative C0 and C1 comparators, respectively (Figure S4E). HG+LPS conditioning prominently induced *Ltf, Chil3*, and *Camp*, while maintaining high *Cd55* expression; in contrast, *Csf3* was not significantly induced. Although *Jun* was also increased, indicating incomplete state specificity, the overall pattern supports partial molecular recapitulation of a C2‐like program rather than complete transcriptional equivalence to endogenous C2 neutrophils. This transcriptional correspondence complements the observed enrichment of surface CD55 in HG+LPS‐conditioned neutrophils and the subsequent enrichment of Ltf in HG+LPS‐derived aNEVs, thereby providing an atlas‐to‐conditioned‐cell‐to‐vesicle validation link for the engineering strategy.

HG+LPS‐derived aNEVs exhibited characteristic small EV dimensions (120–140 nm diameter) distinct from large aging‐neutrophil‐derived vesicles (∼1 µm) [13, 28] (Table 1 and Figure 3G). While LPS alone induced peak EV concentration at 24 h (1.10 × 10^10^ particles mL^−1^), the HG+LPS condition maintained detectable EV production at 48 h (5.80 × 10^9^ particles mL^−1^), indicating that combined metabolic and inflammatory conditioning supports sustained generation of aNEVs under the experimental conditions used (Table 1). BCA analysis showed a higher protein concentration in HG+LPS‐derived aNEV preparations than in control‐derived NEV preparations, reaching 1390.91 ng µL^−1^ at 24 h (Figure 3F). TEM examination confirmed abundant circular, cup‐shaped membranous structures (100–150 nm) with distinct boundaries and characteristic EV ultrastructure (Figure 3B), corroborating NTA size distribution data. Nano‐flow cytometry showed a net CD55‐positive fraction of 11% in HG+LPS‐derived aNEVs (Figure 3C,D). Western blotting detected the EV‐associated proteins TSG101, ALIX, and CD81, together with Ly6G and CD55, in the isolated vesicle preparation (Figure 3E), supporting the characterization of CD55‐bearing neutrophil‐derived EVs.

**TABLE 1:** Nanoparticle tracking analysis of neutrophil‐derived extracellular vesicles under different stimulation conditions.

Sample	Mean diameter (nm)	Peak diameter (nm)	Concentration (particles mL^−1^)
24h‐Control	134.4	120.9	5.10 × 10^9^
24h‐HG	130.5	114.5	2.20 × 10^9^
24h‐LPS	131.9	117.7	1.10 × 10^10^
24h‐HG+LPS	131.1	116.2	5.50 × 10^9^
48h‐Control	128.0	116.3	3.00 × 10^9^
48h‐HG	122.3	109.3	4.40 × 10^9^
48h‐LPS	124.5	114.0	9.60 × 10^9^
48h‐HG+LPS	118.7	106.4	5.80 × 10^9^

**FIGURE 3:**
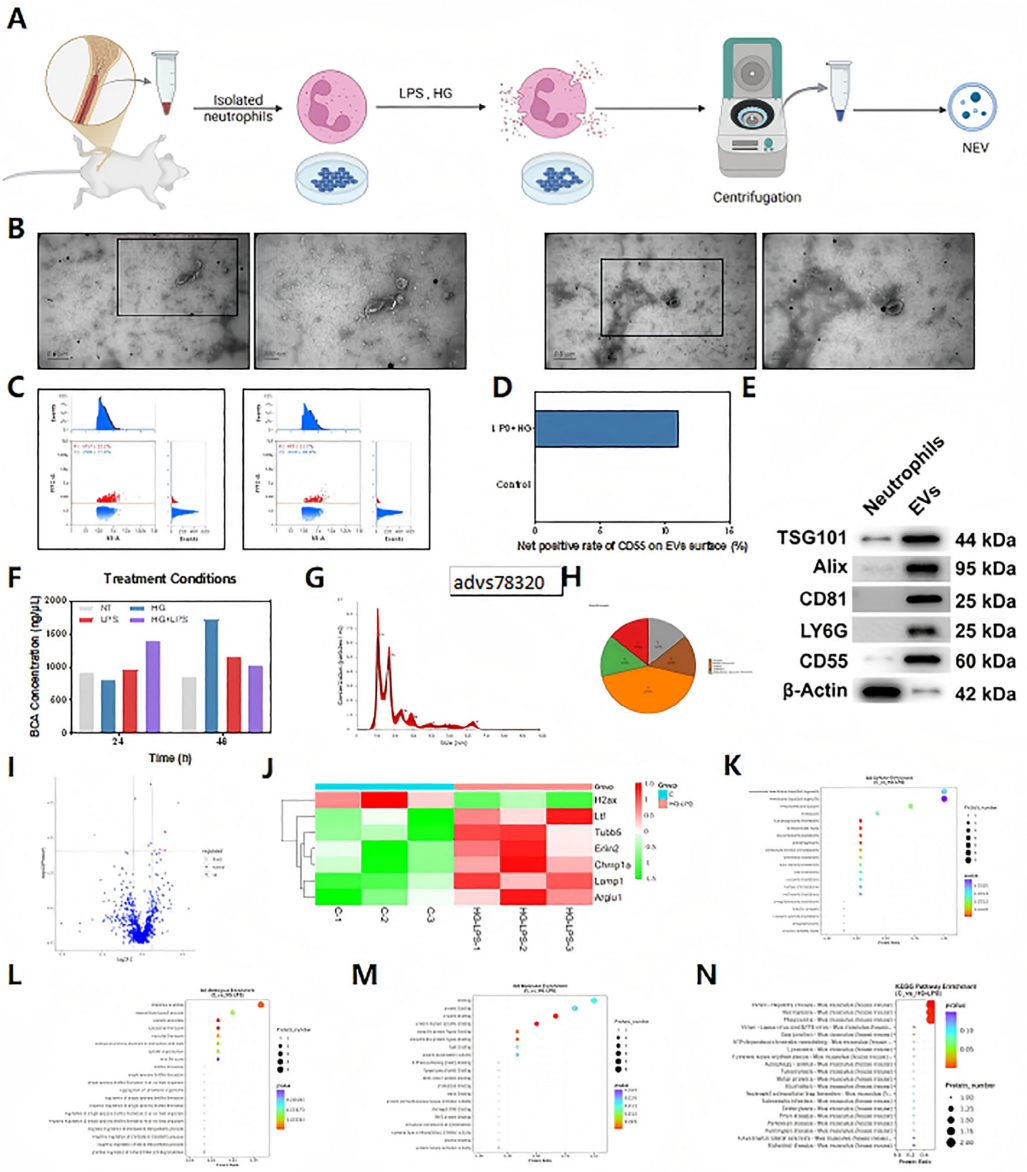
NEVs characterization and proteomics. (A) Isolation workflow. (B) TEM morphology of NEVs under control and HG+LPS conditions. (C, D) Nanoflow cytometry of CD55. (E) Western blot of EV and neutrophil markers. (F) BCA protein quantification. (G) NTA size distribution. (H–N) Proteomic analysis: protein distribution (H), volcano plot (I), heatmap (J), and GO/KEGG enrichment (K–N) (n = 3). The schematic workflow in panel A was created using BioRender.com under a publication license. Created in BioRender. Cui, T. (2026) https://BioRender.com/2nq3bgz.

To examine the interaction of aNEVs with recipient immune cells, PKH67‐labeled aNEVs were co‐cultured with RAW 264.7 macrophages. Confocal microscopy revealed intracellular PKH67‐positive fluorescence, consistent with uptake of labeled vesicular material by recipient macrophages (Figure S5).

To investigate the impact of the diabetic inflammatory microenvironment on NEV protein composition, neutrophils were cultured under either normal conditions or stimulated with high glucose (35 mM) combined with LPS (100 ng mL^−1^) (HG+LPS) for 24 h. Isolated NEVs were subjected to quantitative proteomic analysis (Figure 3H–N).

Quantitative proteomics identified seven differentially abundant proteins between HG+LPS‐derived aNEVs and control‐derived NEVs (fold change ≥ 1.5 or ≤ 0.67, *p* < 0.05), comprising six proteins with increased abundance (LTF, LAMP1, TUBB5, ARGLU1, ERLIN2, and CHMP1A) and one with decreased abundance (H2AX) in HG+LPS‐derived aNEVs (Figure 3I,J). Notably, lactoferrin (Ltf) exhibited the most significant upregulation, consistent with the C2 neutrophil subcluster gene signature. GO/KEGG annotation associated the differentially abundant proteins with biological processes related to antimicrobial defense, DNA repair, cytoskeletal remodeling, and phagosome–autophagy pathways [29, 30, 31, 32] (Figure 3K–N). Notably, Ltf was the most prominently enriched protein and has previously been implicated in the negative regulation of excessive NETosis [33]. Lactoferrin also possesses multiple immunoregulatory properties, including modulation of NF‐κB signaling, LPS neutralization, iron chelation, and complement‐associated regulation [34, 35, 36]. Together, these findings suggest that the altered proteomic profile of HG+LPS‐derived aNEVs may provide complementary molecular features relevant to antimicrobial defense, inflammatory resolution, cellular migration, and redox regulation, although the functional contribution of individual vesicular proteins remains to be directly established. In this context, the molecular enrichment observed in HG+LPS‐derived aNEVs further illustrates the “waste‐to‐treasure” concept by repurposing disease‐relevant neutrophil plasticity into a vesicular product enriched in selected immunoregulatory features.

### 2.3 Gel/aNEV Composite Hydrogel Enables aNEV Encapsulation, Gradual Release, Tissue Adhesion, and Wound‐Mimetic Degradation

Gel/aNEV encapsulation preserved the interconnected porous architecture of the gelatin hydrogel, with pore diameter (112.2 ± 21.0 µm) and porosity (30.37% ± 0.1%) comparable to those of blank Gel (119.1 ± 20.7 µm and 29.82% ± 3.1%, respectively) (Figure 4A–C). Given that the pore size substantially exceeded the hydrodynamic diameter of aNEVs (∼130 nm), the resulting structure is expected to permit vesicle diffusion while supporting physical retention within the crosslinked matrix. The incorporation of aNEVs did not measurably alter the microstructure of the hydrogel. Mechanical characterization further demonstrated that Gel/aNEV retained properties comparable to blank Gel, including a compressive modulus of 3.18 ± 0.29 kPa, a peak compressive stress of 10.55 ± 2.98 kPa, and a strain at failure of 61.89% ± 1.27% (Figure 4D–G). These values fall within the range reported for native soft skin tissues and suggest that aNEV incorporation does not compromise the mechanical integrity required for wound dressing applications [37].

**FIGURE 4:**
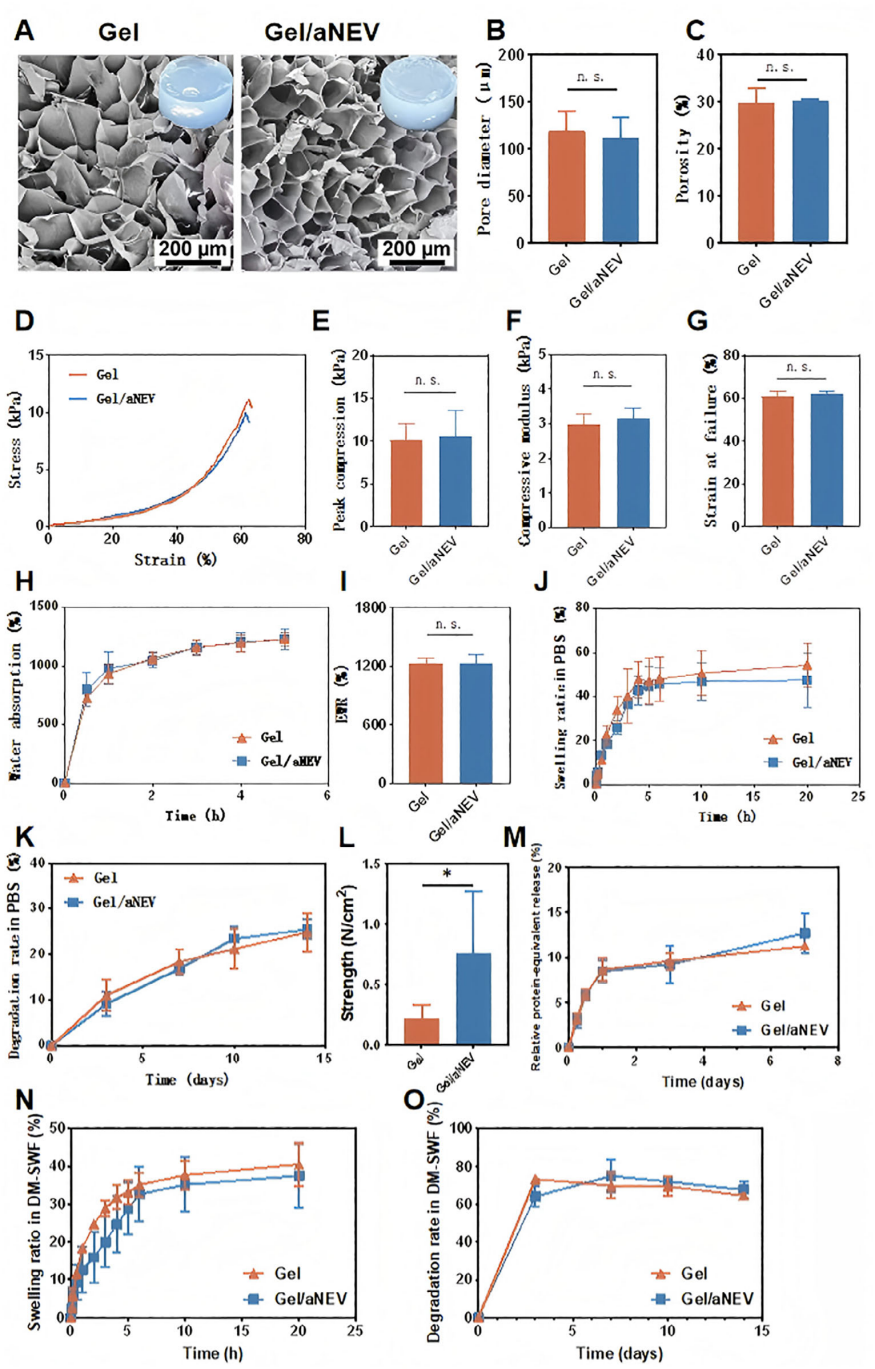
Structural, mechanical, adhesive, release, and wound‐mimetic degradation properties of the Gel/aNEV composite hydrogel. (A) Representative SEM images of Gel and Gel/aNEV hydrogels. Scale bar, 200 µm. (B) Quantification of pore diameter. (C) Hydrogel porosity. (D) Representative compressive stress–strain curves. (E) Peak compressive stress. (F) Compressive modulus. (G) Strain at failure. (H) Water absorption kinetics. (I) Equilibrium water absorption ratio (EWR). (J) Swelling kinetics of Gel and Gel/aNEV in PBS. (K) Degradation profiles in PBS over 14 days. (L) Ex vivo porcine‐skin adhesion strength determined by lap‐shear testing. (M) Relative protein‐equivalent release from Gel and Gel/aNEV over 7 days, determined by BCA assay and expressed as the release‐medium concentration relative to the corresponding initial reference concentration. (N) Swelling kinetics in diabetic microenvironment–mimetic simulated wound fluid (DM‐SWF) over 20 h. (O) Degradation of Gel and Gel/aNEV in DM‐SWF over 14 days. Data are presented as mean ± SD. ^*^ *p* < 0.05, n.s., not significant.

To directly confirm that aNEVs were successfully incorporated into the hydrogel rather than merely mixed with the precursor solution, PKH67‐labeled aNEVs were introduced before mTG‐mediated crosslinking and subsequently visualized by confocal microscopy. PKH67‐positive fluorescent signals were readily detected within the Gel/aNEV matrix, whereas corresponding fluorescence was absent from blank Gel, providing direct visual evidence of successful aNEV incorporation into the crosslinked hydrogel (Figure S6A).

Gel/aNEV exhibited rapid water uptake and a high equilibrium water absorption ratio. Water absorption approached equilibrium within approximately 5 h, reaching 1231.5% ± 86.2%; this endpoint value was also reported as the equilibrium water absorption ratio (EWR) (Figure 4H,I). Such fluid‐handling properties may facilitate exudate absorption and maintenance of a moist wound environment, both of which are favorable for wound management [38]. Dynamic swelling measurements in PBS similarly showed progressive fluid uptake without an evident difference between Gel and Gel/aNEV (Figure 4J), further indicating that incorporation of aNEVs did not substantially alter the bulk hydration behavior of the gelatin network.

Under simplified PBS conditions, Gel and Gel/aNEV displayed comparable degradation profiles, with approximately 25% mass loss after 14 days (Figure 4K). This relatively slow erosion provides a baseline measure of matrix stability in a non‐proteolytic environment [39]. However, because PBS lacks the protein‐rich and proteolytic components characteristic of chronic wound exudate, this experiment was interpreted as a baseline physicochemical assessment rather than a direct representation of hydrogel persistence in diabetic wounds.

In addition to structural stability, maintenance of hydrogel contact with the wound surface is important for localized aNEV delivery. Tissue adhesion was therefore evaluated using an ex vivo porcine‐skin lap‐shear assay (Figure 4L). Gel exhibited an adhesion strength of 0.220 ± 0.117 N cm^−2^, whereas Gel/aNEV reached 0.766 ± 0.514 N cm^−2^ (n = 6). These results demonstrate that incorporation of aNEVs does not compromise the tissue‐contact capability of the mTG‐gelatin matrix and suggest that Gel/aNEV may exhibit enhanced interfacial adhesion. This behavior is consistent with previous reports demonstrating tissue adhesive properties of gelatin–mTG systems under moist biological conditions [40].

The release behavior of the composite hydrogel was evaluated using a time‐course BCA‐based protein‐equivalent assay, with blank Gel analyzed in parallel to characterize gelatin‐derived protein signals (Figure 4M). Relative protein‐equivalent release, expressed as the release‐medium concentration normalized to the corresponding initial reference concentration, increased over time in the Gel/aNEV group, reaching 3.03% ± 0.79%, 5.79% ± 0.50%, 8.45% ± 1.24%, 9.23% ± 2.09%, and 12.72% ± 2.22% at 6 h, 12 h, 24 h, day 3, and day 7, respectively. The corresponding values for blank Gel were 3.23% ± 0.52%, 5.92% ± 0.55%, 8.66% ± 1.25%, 9.61% ± 0.92%, and 11.27% ± 0.24%, with no significant differences between the formulations at the corresponding time points. At 24 h, the baseline‐corrected protein‐equivalent concentrations in the release medium were 600.44 ± 88.28 µg mL^−^^1^ for Gel and 594.10 ± 92.42 µg mL^−^^1^ for Gel/aNEV (*p* = 0.848). These results indicate comparable relative protein‐equivalent release profiles for Gel and Gel/aNEV under the tested conditions. Because gelatin‐derived proteins and peptides contribute to the BCA signal, this assay characterizes composite protein‐equivalent release rather than EV‐specific release. Previous studies have reported prolonged retention and gradual release of incorporated EVs from transglutaminase‐crosslinked protein hydrogels, supporting the use of enzymatically crosslinked protein matrices as local EV‐delivery depots [41, 42].

To evaluate hydrogel behavior under conditions more closely reflecting the biochemical environment of diabetic wound exudate, swelling and degradation were further examined in diabetic microenvironment–mimetic simulated wound fluid (DM‐SWF) containing 25 mM glucose and 200 ng mL^−1^ active MMP‐9 at 37°C. Protein‐containing simulated wound fluids have been proposed as more clinically relevant media for evaluating wound‐dressing fluid handling than simple saline‐based solutions [43].

Both Gel and Gel/aNEV progressively absorbed DM‐SWF during the 20‐h observation period (Figure 4N). At 20 h, the swelling ratios reached 40.52% ± 5.71% and 37.53% ± 8.36%, respectively, with no significant difference between the two formulations. These findings indicate that incorporation of aNEVs does not substantially alter the fluid‐uptake behavior of the gelatin matrix under diabetic wound‐mimetic conditions.

The influence of the wound‐mimetic environment was more evident in the degradation profile. In contrast to the limited degradation observed in PBS, exposure to DM‐SWF markedly increased matrix erosion (Figure 4O). At day 14, degradation reached approximately 64.8% for Gel and 67.8% for Gel/aNEV, indicating comparable susceptibility of the two formulations to the proteinase‐containing environment. Thus, PBS alone appears to overestimate the persistence of the gelatin matrix under wound‐relevant proteolytic conditions. The inclusion of active MMP‐9 is biologically relevant because elevated MMP‐9 abundance and activity have been directly detected in chronic and diabetic wound fluids [44].

These wound‐mimetic results also refine the interpretation of the increased *Mmp9* expression observed in Gel/aNEV‐treated wounds in vivo (Figure 9E). Rather than establishing a direct causal MMP‐9‐triggered release mechanism, the combined findings indicate that the gelatin matrix is susceptible to enhanced degradation in a protease‐rich environment and that local proteolytic activity may therefore contribute to matrix remodeling and aNEV availability during wound healing.

Given the substantial matrix erosion observed under DM‐SWF conditions, we next asked whether nanoscale particles within the characteristic aNEV size range remained detectable in the residual hydrogel after prolonged incubation. Residual Gel and Gel/aNEV samples recovered after 14 days of incubation in DM‐SWF were subjected to NTA to assess the persistence of nanoscale particles during prolonged matrix degradation (Figure S6B). Blank Gel itself generated a predominantly smaller nanoscale particle population, with a mean diameter of 82.1 ± 3.1 nm, a D50 of 67.6 ± 1.7 nm, and a particle concentration of 1.03 × 10^10^ ± 7.24 × 10^8^ particles mL^−1^. These signals likely reflect, at least in part, gelatin‐derived colloidal or degradation‐associated particles generated during prolonged incubation. In contrast, residual Gel/aNEV displayed a broader particle‐size distribution shifted toward the characteristic size range of aNEVs, with a mean diameter of 140.0 ± 8.1 nm, a D50 of 119.0 ± 7.4 nm, and a D90 of 236.6 ± 21.6 nm. The total particle concentration reached 3.24 × 10^10^ ± 7.99 × 10^9^ particles mL^−1^, approximately threefold higher than that detected in blank Gel. Importantly, the central particle‐size distribution in residual Gel/aNEV remained close to that of freshly prepared aNEVs (∼130 nm), supporting the persistence of an aNEV‐sized nanoparticle population after 14 days of exposure to the wound‐mimetic environment.

Because NTA detects light‐scattering nanoparticles without providing biochemical identity, the detected population cannot be assigned exclusively to intact aNEVs. In particular, smaller particles and the broader high‐size tail may include gelatin‐derived colloids, degradation products, or particle aggregates. Accordingly, these findings are interpreted as evidence for the physical persistence of an aNEV‐sized particle population within the degrading Gel/aNEV matrix, rather than as direct evidence that all detected particles represent intact or biologically active aNEVs [41, 42].

Collectively, these orthogonal analyses establish successful aNEV incorporation, gradual composite release, measurable tissue adhesion, preserved fluid uptake, protease‐sensitive matrix degradation, and persistence of an aNEV‐sized nanoparticle population after prolonged exposure to a diabetic wound‐mimetic environment. These features collectively support the Gel/aNEV matrix as a localized delivery platform that undergoes progressive wound‐associated remodeling while retaining vesicle‐sized nanoparticles over the healing period.

### 2.4 Biocompatible Gel/aNEV Hydrogel Promotes Cell Migration

The cytocompatibility of Gel and Gel/aNEV extracts was evaluated using HUVECs. Live/dead staining demonstrated predominantly viable cells with minimal dead‐cell signals throughout the 3‐day observation period in all treatment groups (Figure 5A). Quantitative CCK‐8 analysis showed a time‐dependent increase in metabolic activity (Figure 5C), while calculated cell survival rates remained above 90% after 1 and 3 days, with no significant differences relative to untreated controls (Figure 5D). These findings indicate that neither Gel nor Gel/aNEV exerted detectable cytotoxic effects under the experimental conditions tested.

**FIGURE 5:**
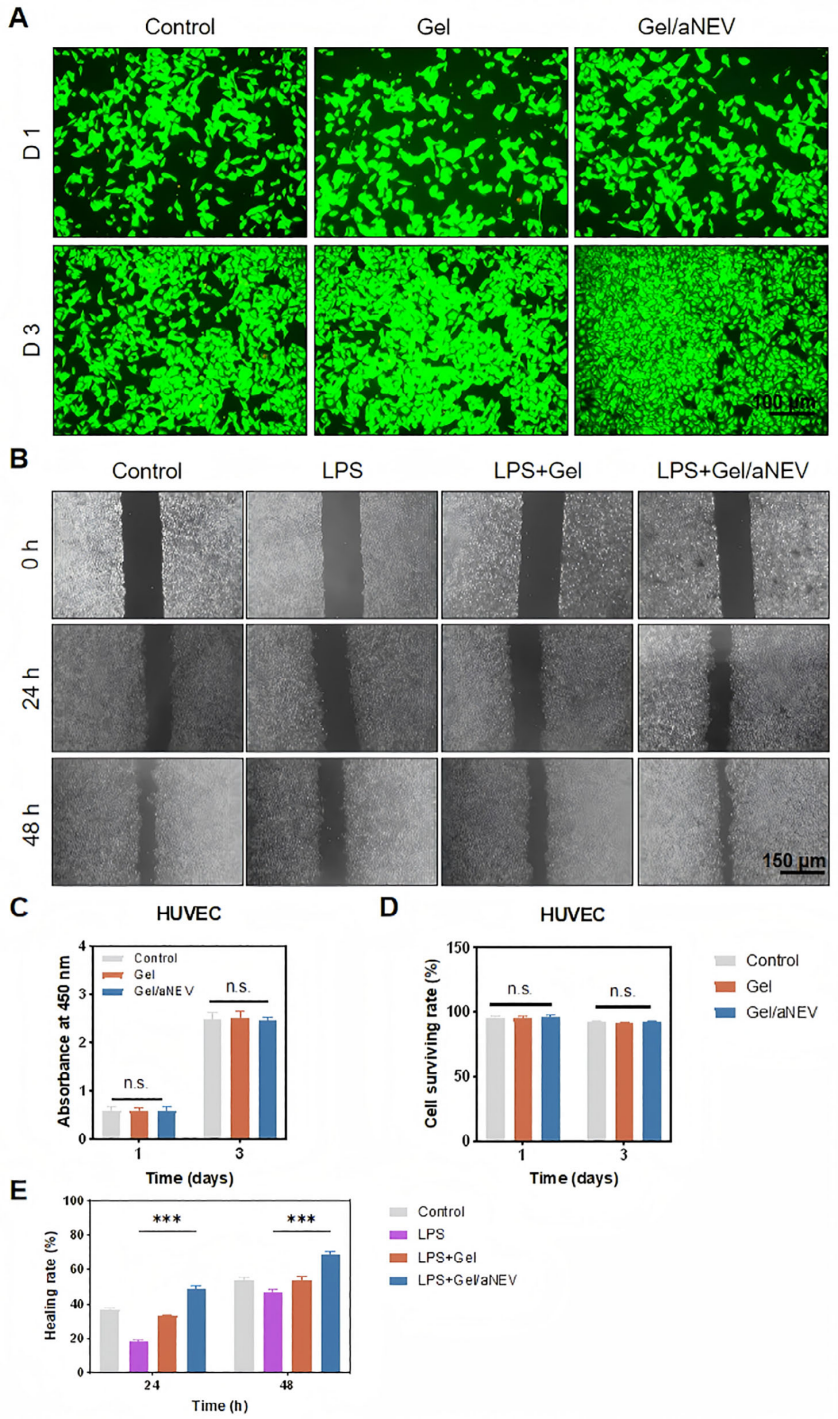
Biocompatibility and pro‐migratory effects of Gel/aNEV. (A) Live/dead staining of HUVECs after 1 and 3 days of co‐culture with hydrogel extracts (Control, Gel, and Gel/aNEV). Green: calcein‐AM (live cells); red: propidium iodide (dead cells). Scale bar: 100 µm. (B) Scratch assay images at 0, 24, and 48 h showing cell migration under different conditions (Control, LPS, LPS + Gel, and LPS + Gel/aNEV). Scale bar: 150 µm. (C) CCK‐8 assay quantifying cell viability at 450 nm. (D) Cell survival rate. (E) Quantification of wound closure rate. Data are mean ± SD (n = 3). ^*^ *p* < 0.05, ^**^ *p* < 0.01, ^***^ *p* < 0.001.

To assess the effects of Gel/aNEV on endothelial cell migration under inflammatory conditions, scratch assays were performed using HUVECs exposed to LPS. Simultaneous treatment with Gel/aNEV increased scratch closure compared with LPS alone at both 24 and 48 h (*p* < 0.001; Figure 5B,E). These findings support the ability of Gel/aNEV to promote endothelial cell migration under inflammatory conditions.

### 2.5 Gel/aNEV Suppresses Inflammatory Activation and Promotes Reparative Macrophage Reprogramming

Macrophages serve as pivotal orchestrators of wound healing, exhibiting remarkable phenotypic plasticity that governs inflammatory resolution and tissue repair [45]. Upon tissue injury, circulating monocytes are recruited to the wound site and differentiate into pro‐inflammatory M1 macrophages, which phagocytose debris and pathogens while secreting pro‐inflammatory cytokines (TNF‐α, IL‐1β, IL‐6) to initiate host defense [46]. However, persistent M1 polarization contributes to chronic inflammation and impaired healing, particularly in diabetic wounds [47]. In diabetic foot ulcers, macrophages display dysfunctional polarization dynamics characterized by delayed M1‐to‐M2 transition, sustained pro‐inflammatory phenotype, and impaired efferocytosis capacity, resulting in prolonged inflammation and stalled repair [48]. Hyperglycemia‐induced metabolic reprogramming and advanced glycation end product accumulation further exacerbate macrophage dysfunction, creating a self‐perpetuating cycle of inflammation and tissue damage [49]. Timely transition to the anti‐inflammatory M2 phenotype is therefore critical for wound resolution; M2 macrophages promote angiogenesis, collagen deposition, and extracellular matrix remodeling through secretion of IL‐4, IL‐10, and TGF‐β, thereby accelerating re‐epithelialization and tissue regeneration [50]. Restoration of physiological macrophage polarization dynamics has emerged as a promising therapeutic strategy for refractory diabetic wounds [51].

We employed flow cytometry, immunofluorescence, and molecular profiling to characterize macrophage responses to Gel/aNEV treatment (Figure 6). Flow cytometric analysis of RAW 264.7 macrophages showed that Gel/aNEV treatment markedly increased the proportion of CD206^+^ cells compared with LPS stimulation alone. In the direct LPS vs. LPS+Gel/aNEV comparison, CD86^+^ cell frequencies were not significantly different (Figure 6A,B). Consistent with these findings, immunofluorescence analysis demonstrated reduced iNOS expression and enhanced CD206 expression in CD68^+^ macrophages following Gel/aNEV treatment (Figure 6C).

**FIGURE 6:**
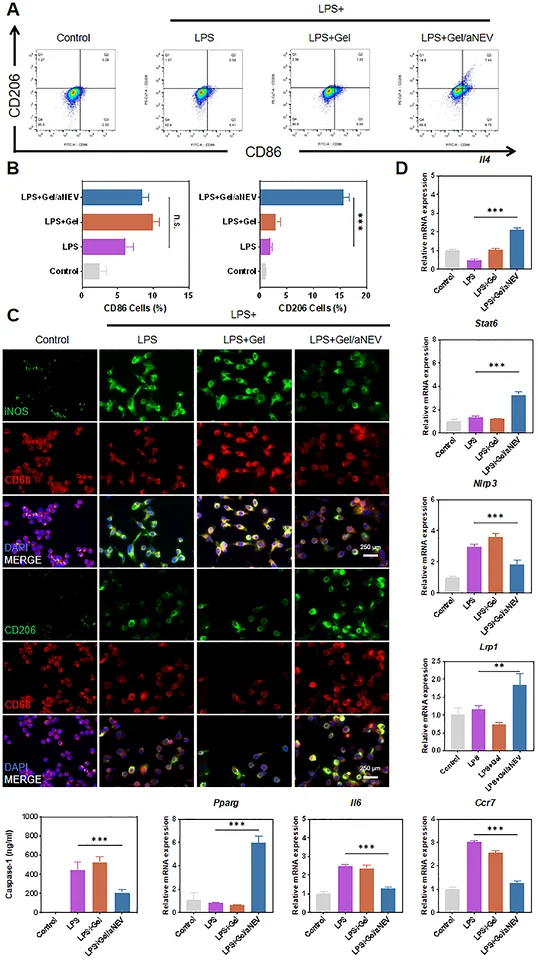
Gel/aNEV suppresses inflammatory activation and promotes reparative macrophage reprogramming. (A) Representative flow‐cytometry plots of CD86 and CD206 expression in RAW 264.7 macrophages from the Control, LPS, LPS+Gel, and LPS+Gel/aNEV groups. (B) Quantification of CD86^+^ and CD206^+^ cell populations. (C) Representative immunofluorescence images of iNOS/CD68 and CD206/CD68 staining. Nuclei were counterstained with DAPI. Scale bars are indicated in the images. (D) RT‐qPCR analysis of macrophage phenotype‐ and inflammation‐associated genes, including *Il4, Stat6, Nlrp3, Lrp1, Pparg, Il6*, and *Ccr7*. Caspase‐1 levels were additionally quantified as an inflammasome‐associated readout. Gel/aNEV treatment was associated with increased expression of reparative macrophage‐related genes and reduced inflammatory and NLRP3/Caspase‐1‐associated responses. Data are presented as mean ± SD (n = 3). ^*^ *p* < 0.05, ^**^ *p* < 0.01, ^***^ *p* < 0.001; n.s., not significant.

The expanded molecular analysis further revealed coordinated suppression of inflammatory activation and induction of reparative signaling. Gel/aNEV markedly reduced the expression of *Ccr7* and *Il6*, while simultaneously suppressing the inflammasome‐associated gene *Nlrp3*. Consistently, Caspase‐1 levels were substantially reduced following Gel/aNEV treatment (Figure 6D). Because NLRP3 inflammasome assembly activates Caspase‐1 and promotes inflammatory cytokine maturation, the coordinated reduction in *Nlrp3* and Caspase‐1 supports attenuation of an NLRP3–Caspase‐1‐associated inflammatory program [52].

Conversely, Gel/aNEV markedly increased *Il4, Stat6*, and *Pparg* expression. STAT6 and PPARγ are closely linked to alternative macrophage activation and inflammatory resolution, and STAT6‐dependent PPARγ activation has been shown to facilitate pro‐resolving macrophage programming and efferocytic activity [53]. The concurrent increase in *Il4*, *Stat6*, and *Pparg* is therefore consistent with a broader reparative macrophage‐associated transcriptional response rather than changes restricted to individual M1/M2 markers.

Notably, *Lrp1* expression was also markedly increased in Gel/aNEV‐treated macrophages. Proteomic analysis identified enrichment of lactoferrin (Ltf) within HG+LPS‐derived aNEVs, a multifunctional immunoregulatory protein previously associated with modulation of inflammatory signaling through iron sequestration, LPS neutralization, and regulation of inflammatory responses [54]. LRP1 is a recognized endocytic and signaling receptor capable of binding lactoferrin, and lactoferrin‐based nanocarriers have been shown to target LRP1‐expressing activated macrophages [55, 56]. The concomitant enrichment of Ltf in aNEVs and induction of *Lrp1* in recipient macrophages therefore points to a potential Ltf–LRP1‐associated interface linking vesicular cargo delivery with macrophage immunomodulation.

These cargo‐associated molecular changes coincided with reduced *Nlrp3* and Caspase‐1 levels and increased expression of *Il4*, *Stat6*, and *Pparg*, suggesting a potential relationship between the vesicular cargo profile and macrophage immunoregulatory responses. In addition, neutrophil‐derived extracellular vesicles have been reported to contain other immunoregulatory constituents, including metabolites such as itaconate, that can modulate macrophage inflammatory activity through metabolic mechanisms [13, 57]. Collectively, these observations are consistent with a multicomponent model in which vesicular cargo may complement membrane‐associated signals in shaping macrophage responses. Direct perturbation of individual cargo components will be required to establish their relative causal contributions.

### 2.6 Gel/aNEV Rebalances Complement Activation, Regulation, and Anaphylatoxin Signaling

The complement system serves as a critical component of innate immunity, playing a dual role in wound healing through pathogen opsonization and inflammatory regulation [58]. Upon tissue injury, complement activation generates the anaphylatoxins C3a and C5a, which interact with C3aR and C5aR1/C5aR2, respectively, on immune cells and participate in the regulation of inflammatory responses [59]. While essential for host defense, excessive or persistent complement activation perpetuates chronic inflammation and impairs wound resolution, particularly in diabetic wounds where hyperglycemia‐driven complement dysregulation exacerbates tissue damage [60]. Therapeutic modulation of the complement cascade has emerged as a promising strategy to restore immune homeostasis and accelerate healing in refractory wounds [61].

To comprehensively evaluate complement‐associated regulation by Gel/aNEV, we examined complement activation, anaphylatoxin receptor signaling, endogenous complement regulation, inflammatory cytokine production, and oxidative stress (Figure 7). ELISA showed that C3a and IL‐6 levels were lower in the LPS+Gel/aNEV group than in the LPS group (Figure 7B). However, the two mediators exhibited different responses relative to the blank‐hydrogel condition: IL‐6 levels were lower in the LPS+Gel/aNEV group than in the LPS + Gel group, whereas C3a levels were higher. These findings support attenuation of complement‐associated and inflammatory responses relative to LPS stimulation alone.

**FIGURE 7:**
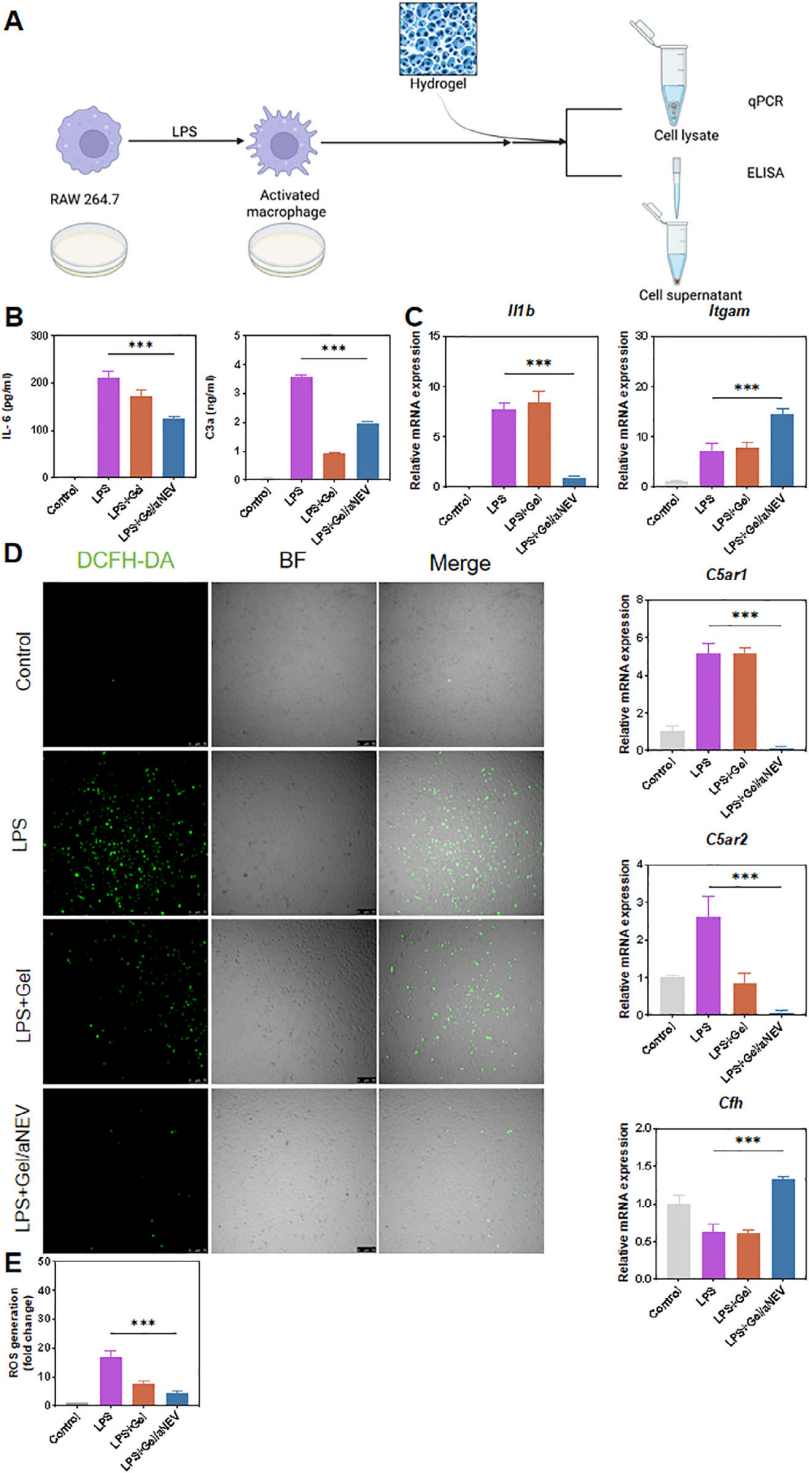
Gel/aNEV rebalances complement‐associated inflammatory signaling and attenuates oxidative stress in activated macrophages. (A) Schematic illustration of the in vitro experimental design. RAW 264.7 macrophages were activated with LPS and treated with Gel or Gel/aNEV. Cells and culture supernatants were collected for RT‐qPCR and ELISA analyses, respectively. (B) ELISA quantification of IL‐6 and C3a levels in macrophage culture supernatants. (C) RT‐qPCR analysis of *Il1b, C5ar2, Cfh, C5ar1*, and *Itgam*. Gel/aNEV treatment reduced expression of pro‐inflammatory and complement receptor‐associated genes while increasing *Cfh* and *Itgam*, consistent with rebalancing of complement‐associated immune signaling. (D) Representative DCFH‐DA fluorescence images showing intracellular ROS production in the Control, LPS, LPS+Gel, and LPS+Gel/aNEV groups. Corresponding bright‐field and merged images are shown. Scale bars are indicated in the images. (E) Quantification of intracellular ROS generation. Data are presented as mean ± SD (n = 3). ^*^ *p* < 0.05, ^**^ *p* < 0.01, ^***^ *p* < 0.001, n.s., not significant. The experimental schematic in panel A was created using BioRender.com under a publication license. Created in BioRender. Cui, T. (2026) https://BioRender.com/gheggd6.

Compared with the LPS and LPS + Gel groups, the LPS + Gel/aNEV group exhibited lower expression of *Il1b*, *C5ar1*, and *C5ar2* (Figure 7C). In contrast, expression of *Cfh*, which encodes the endogenous complement regulator factor H, was higher in the LPS + Gel/aNEV group. Expression of *Itgam*, encoding the CD11b subunit of complement receptor 3 (CR3), was also increased. These transcriptional changes were accompanied by the differences in soluble inflammatory and complement‐associated mediators described above.

These changes are particularly relevant to the enrichment of CD55 on the aNEV membrane. CD55 is a GPI‐anchored complement regulator that accelerates the decay of C3 and C5 convertases and thereby constrains complement amplification. Compared with LPS stimulation alone, Gel/aNEV treatment was associated with lower C3a levels, reduced *C5ar1*/*C5ar2* expression, and increased *Cfh* expression. C3a levels in the Gel/aNEV group were intermediate between those in the LPS and LPS + Gel groups.

Complement activation and oxidative stress are also closely interconnected during chronic inflammation [62]. Consistent with this relationship, DCFH‐DA staining showed robust ROS accumulation after LPS stimulation, whereas Gel/aNEV markedly reduced intracellular ROS generation (Figure 7D,E). The parallel reductions in C3a, IL‐6, and ROS indicate that attenuation of excessive complement signaling coincides with suppression of downstream inflammatory and oxidative amplification.

Together with the macrophage‐reprogramming effects observed in Figure 6, these findings support two complementary immunoregulatory dimensions of aNEVs: a membrane‐associated complement‐regulatory component centered on CD55, and a cargo‐associated macrophage regulatory component involving Ltf and other bioactive vesicular constituents. Their convergence is associated with suppression of complement‐driven inflammatory amplification and establishment of a pro‐resolving macrophage state.

### 2.7 A Single Application of Gel/aNEV Accelerates Diabetic Wound Healing and is Associated With Immune Microenvironment Reprogramming

To evaluate the therapeutic efficacy of aNEV‐loaded hydrogel (Gel/aNEV) in chronic wound healing, a streptozotocin‐induced diabetic mouse model with full‐thickness excisional wounds was established, and animals were randomly assigned to the Control, Gel, and Gel/aNEV groups. As shown in Figure 8, Gel/aNEV‐treated wounds exhibited significantly accelerated closure over the 14‐day observation period compared with both the Control and Gel groups. Quantitative analysis of wound closure showed that, by postoperative day 3, wound healing rates were 32.38% ± 3.24%, 17.19% ± 4.03%, and 16.48% ± 6.12% in the Gel/aNEV, Control, and Gel groups, respectively (Figure 8B,C). This pro‐healing advantage persisted throughout the observation period. By day 14, wounds in the Gel/aNEV group were almost completely re‐epithelialized, whereas both Control and Gel groups displayed substantial unhealed areas.

**FIGURE 8:**
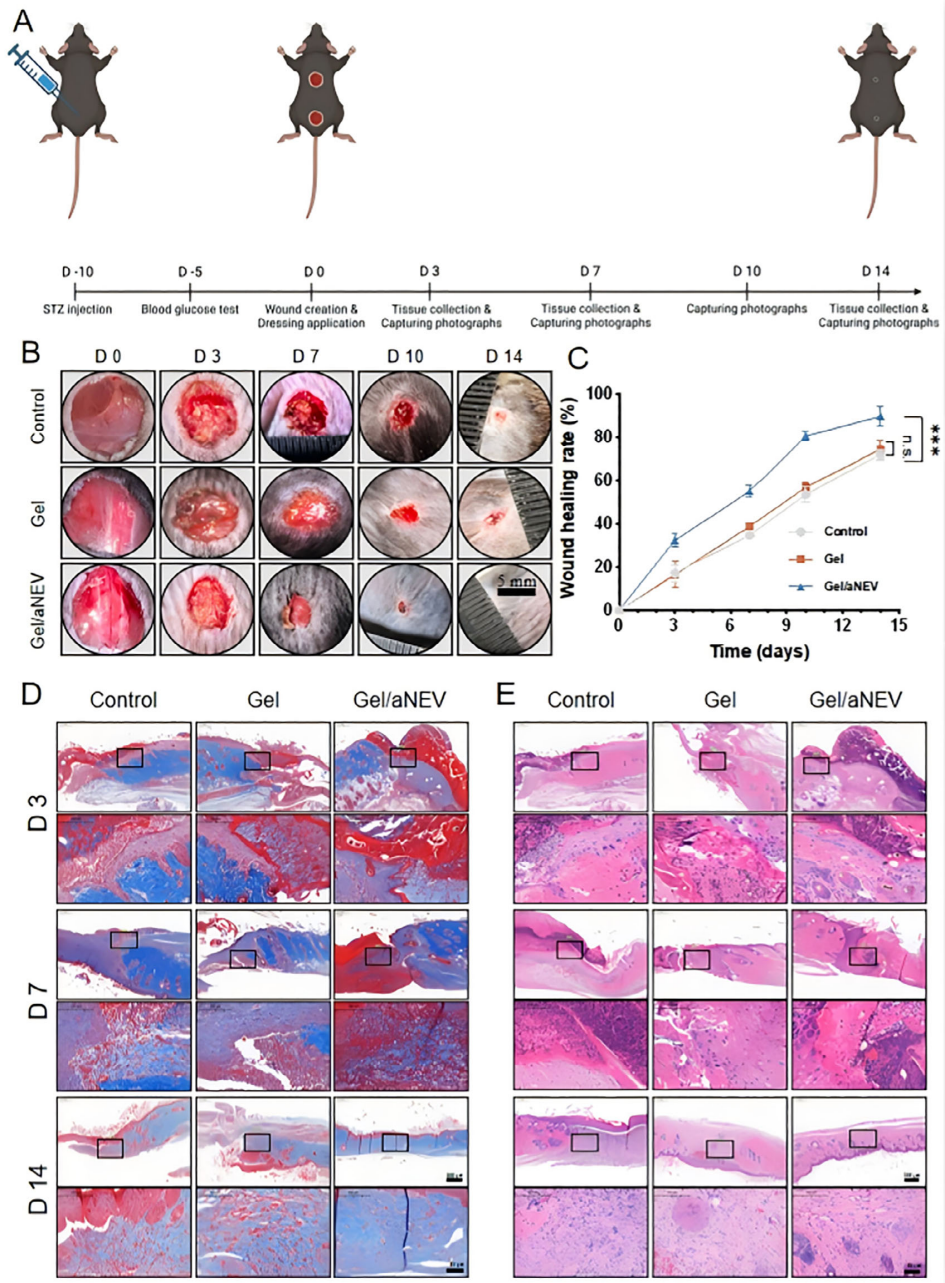
Therapeutic efficacy of Gel/aNEV in diabetic wound healing. (A) Experimental timeline: STZ injection, blood glucose monitoring, wound creation, hydrogel treatment, and tissue harvest. (B) Macroscopic wound appearance. (C) Wound healing rate (%) (n = 3 per group). (D) Masson's trichrome staining showing collagen maturation. (E) H&E staining showing granulation tissue formation and re‐epithelialization. Scale bars are indicated in the images. Data are mean ± SD. ^*^ *p* < 0.05, ^**^ *p* < 0.01, ^***^ *p* < 0.001. The experimental timeline in panel A was created using BioRender.com under a publication license. Created in BioRender. Cui, T. (2026) https://BioRender.com/e2i4g2w.

Hematoxylin and eosin (H&E) staining of tissue sections from days 7 and 14 (Figure 8E) demonstrated that Gel/aNEV treatment substantially improved tissue regeneration quality. Compared to controls, the Gel/aNEV group exhibited more abundant granulation tissue, better‐organized neovascularization, and reduced inflammatory cell infiltration at day 7. By day 14, complete re‐epithelialization was achieved in the Gel/aNEV group, with early skin appendage structures visible. Masson's trichrome staining (Figure 8D) further showed denser and more organized collagen deposition in Gel/aNEV‐treated wounds than in the Control and Gel groups at days 7 and 14.

To characterize immune and microenvironmental changes associated with Gel/aNEV‐mediated wound repair, we evaluated inflammatory cytokine profiles and immune‐cell phenotypes during the critical healing period using immunohistochemical and immunofluorescence analyses. We hypothesized that the observed therapeutic responses may involve interactions among the inflammatory wound microenvironment, hydrogel remodeling, and aNEV‐associated immunoregulatory signals. Elevated proteolytic activity characteristic of diabetic wounds may facilitate gelatin‐matrix degradation and local aNEV release, whereas membrane‐associated CD55 and vesicular cargos such as lactoferrin may contribute to modulation of excessive complement‐associated and inflammatory signaling. Dysregulated inflammation, characterized by persistent neutrophil accumulation, impaired macrophage phenotypic transition, and sustained production of pro‐inflammatory mediators, is a hallmark of chronic diabetic wounds [6]. Accordingly, modulation of these inflammatory and immune responses provides a biologically relevant framework for interpreting the wound‐healing effects observed following Gel/aNEV treatment [63].

Immunohistochemical staining and quantitative analysis revealed distinct temporal patterns of inflammatory cytokine expression (Figure 9A,B). Mean IL‐1β staining intensity decreased from day 3 to day 7 across all groups, whereas mean IL‐10 staining intensity increased over this interval. In contrast, mean TNF‐α staining intensity increased in the Control and Gel groups but decreased in the Gel/aNEV group. At both assessed time points, Gel/aNEV‐treated wounds exhibited lower TNF‐α and IL‐1β staining intensities and higher IL‐10 staining intensity than the Control and Gel groups, consistent with a more anti‐inflammatory local tissue profile.

**FIGURE 9:**
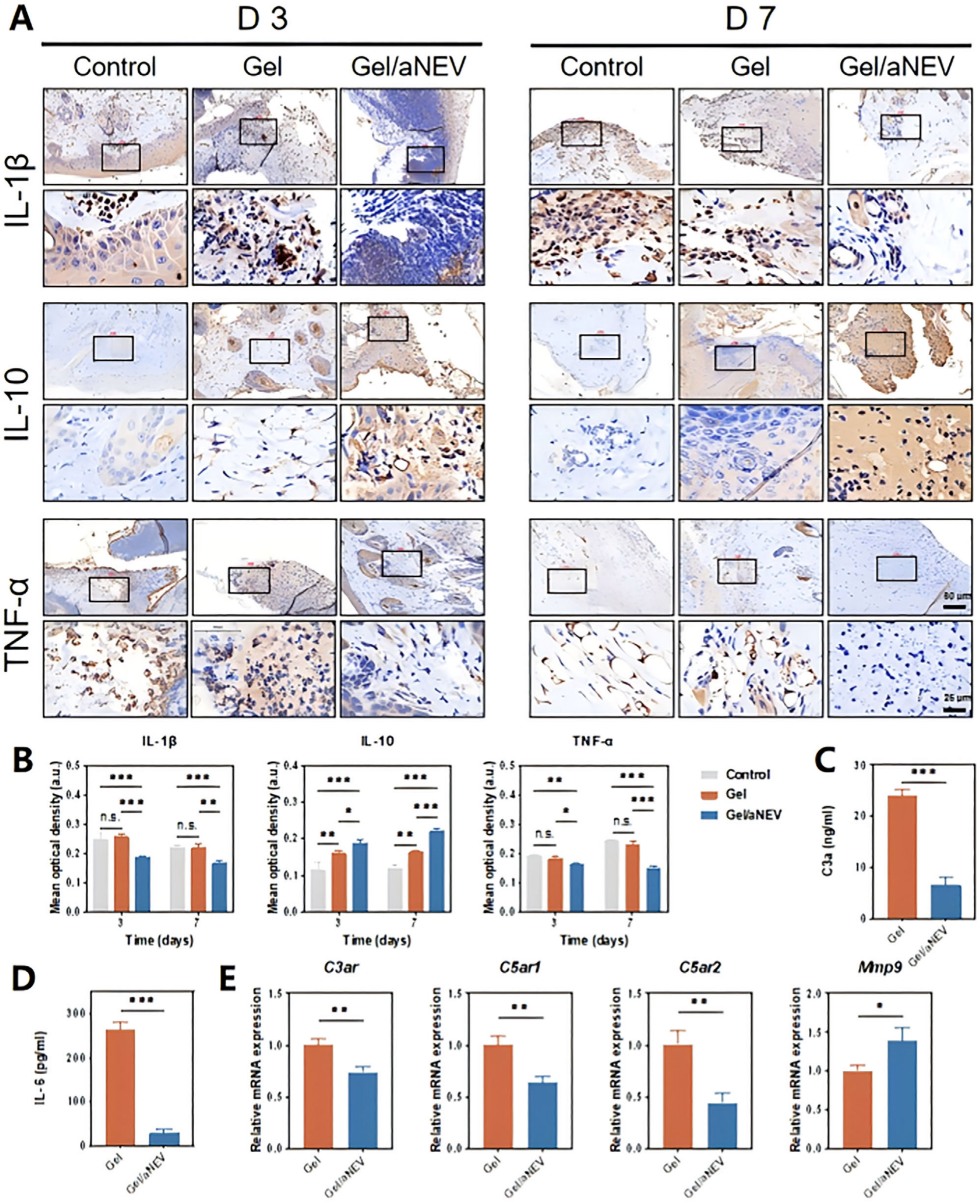
Gel/aNEV treatment is associated with reduced inflammatory and complement‐associated responses in diabetic wounds. (A) Representative immunohistochemical staining of IL‐1β, IL‐10, and TNF‐α in wound tissues from Control, Gel, and Gel/aNEV groups at days 3 and 7. Boxed regions are shown at higher magnification. Scale bars: 60 µm for low‐magnification images and 25 µm for enlarged images. (B) Quantification of the mean optical density of IL‐1β, IL‐10, and TNF‐α immunohistochemical staining at days 3 and 7. (C) C3a levels in wound tissues measured by ELISA. (D) IL‐6 levels in wound tissues measured by ELISA. (E) RT‐qPCR analysis of complement‐associated genes *C3ar1*, *C5ar1*, and *C5ar2*, together with the tissue‐remodeling gene *Mmp9*, in Gel‐ and Gel/aNEV‐treated wounds. Data are presented as mean ± SD (n = 3 per group). ^*^ *p* < 0.05, ^**^ *p* < 0.01, ^***^ *p* < 0.001; n.s., not significant.

Immunofluorescence analysis further revealed dynamic remodeling of the wound immune microenvironment following Gel/aNEV treatment (Figure 10A,B). At day 3, Gel/aNEV‐treated wounds showed a transient increase in Ly6G‐positive area, which markedly declined by day 7, indicating an early Ly6G‐associated response followed by subsequent resolution. Because CD55 staining was also observed in non‐Ly6G tissue structures, including hair‐associated regions, total tissue CD55 positivity was not considered a sufficiently specific measure of neutrophil‐associated CD55. Accordingly, CD55 was quantified as the proportion of Ly6G‐positive staining co‐expressing CD55 (CD55^+^Ly6G^+^/Ly6G^+^). This fraction was significantly increased in the Gel/aNEV group at both days 3 and 7, supporting enhanced association of CD55 with Ly6G‐positive signals during the early healing phase. In parallel, Gel/aNEV‐treated wounds exhibited increased CD206‐positive staining together with reduced CD86‐positive staining at both time points, consistent with remodeling toward a more reparative immune phenotype.

**FIGURE 10:**
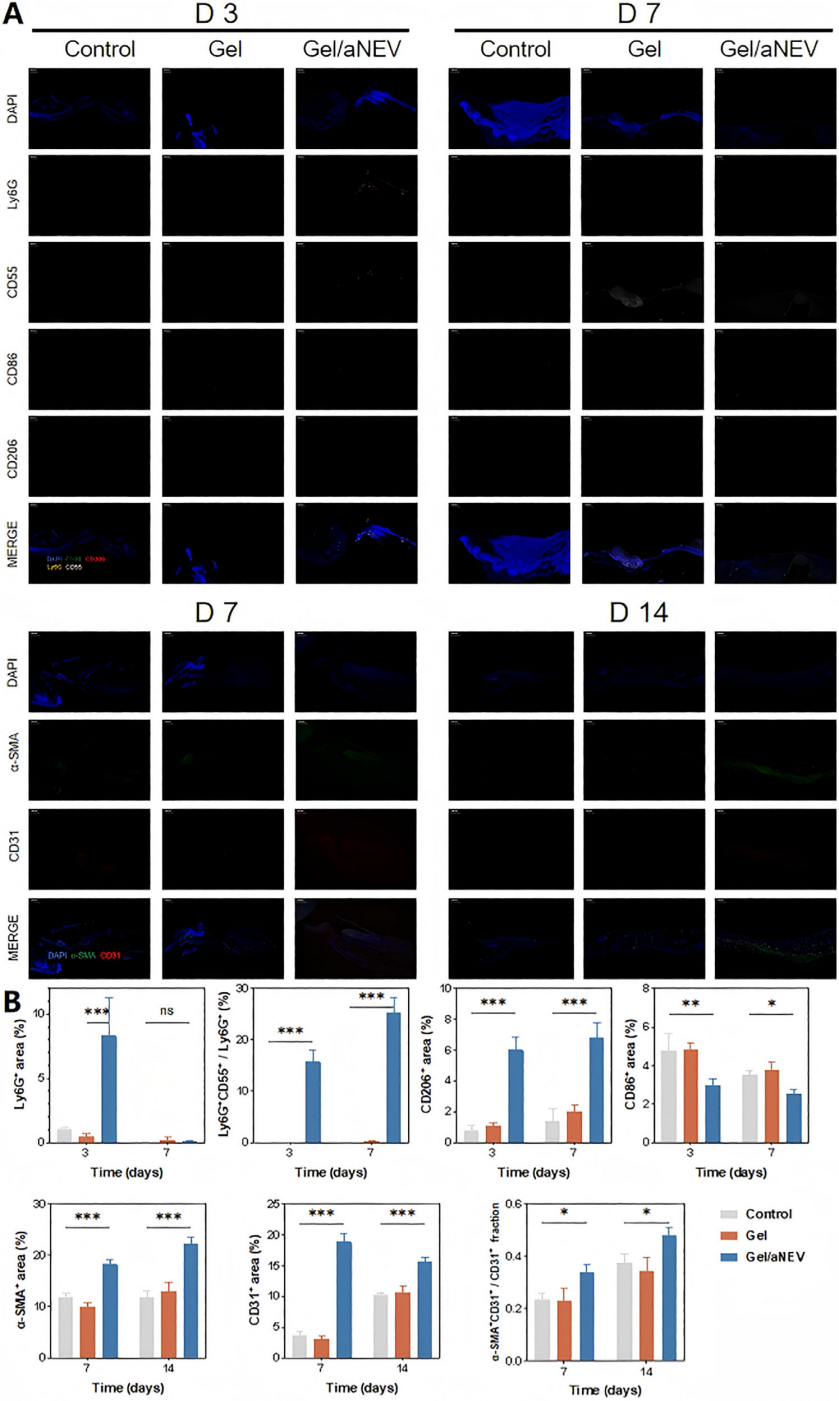
Immunofluorescence analysis of immune‐associated and vascular responses in Gel/aNEV‐treated diabetic wounds. (A) Representative immunofluorescence images showing individual fluorescence channels and corresponding merged images. DAPI, Ly6G, CD55, CD86, and CD206 staining were evaluated at days 3 and 7, whereas CD31 and α‐SMA staining were evaluated at days 7 and 14. (B) Quantification of Ly6G‐positive area, CD55^+^Ly6G^+^/Ly6G^+^ fraction, CD206‐positive area, CD86‐positive area, α‐SMA‐positive area, CD31‐positive area, and α‐SMA^+^CD31^+^/CD31^+^ fraction. Because CD55 signal was also detected in non‐Ly6G tissue structures, neutrophil‐associated CD55 was quantified specifically within Ly6G‐positive regions rather than using total tissue CD55‐positive area. The α‐SMA^+^CD31^+^/CD31^+^ fraction was used to evaluate α‐SMA association with CD31‐positive vascular structures. Data are presented as mean ± SD (n = 3 per group). ^*^ *p* < 0.05, ^**^ *p* < 0.01, ^***^ *p* < 0.001; n.s., not significant.

To assess vascular responses associated with Gel/aNEV treatment, immunofluorescence staining was performed at days 7 and 14 to quantify CD31^+^ and α‐smooth muscle actin (α‐SMA)^+^ areas, together with the fraction of CD31^+^ staining associated with α‐SMA positivity (α‐SMA^+^CD31^+^/CD31^+^; Figure 10) [64]. Impaired neovascularization, characterized by reduced capillary density, poor vessel maturation, and insufficient pericyte recruitment, represents a critical pathological feature of diabetic wounds that perpetuates tissue hypoxia and delays repair [65]. Therapeutic promotion of functional angiogenesis with stable, mature vessels is therefore essential for restoring tissue perfusion and accelerating wound closure in chronic wounds [66].

Compared with Control and Gel groups, Gel/aNEV‐treated wounds exhibited increased CD31‐positive and α‐SMA‐positive areas at days 7 and 14. Moreover, the proportion of CD31‐positive vascular structures associated with α‐SMA staining (α‐SMA^+^CD31^+^/CD31^+^) was increased, consistent with enhanced vascularization and a more mature vascular phenotype. Together with the observed changes in inflammatory and immune‐cell‐associated markers, these findings support coordinated immune remodeling and vascular responses during Gel/aNEV‐treated diabetic wound repair.

### 2.8 In Vivo Assessment of Complement‐Associated and Inflammatory Responses

Compared with the Gel group, Gel/aNEV treatment significantly reduced the expression of the complement‐associated receptors C3ar1, C5ar1, and C5ar2 (Figure 9E), while ELISA revealed lower tissue levels of C3a and IL‐6 (Figure 9C,D). Together, these findings are consistent with reduced complement‐associated inflammatory activity in Gel/aNEV‐treated diabetic wounds. *Mmp9* was additionally evaluated as an extracellular matrix‐remodeling marker (Figure 9E). Because elevated proteolytic activity, particularly MMP‐9 activity, is a recognized feature of chronic diabetic wounds, the gelatin matrix may undergo enhanced degradation within such a wound environment. These observations, together with the protease‐sensitive degradation demonstrated under wound‐mimetic conditions, are consistent with a potential role for local proteolytic remodeling in facilitating aNEV release, although an MMP‐9‐dependent release mechanism was not directly established in vivo.

### 2.9 Integrated Transcriptomic Analysis Reveals Coordinated Immune Regulation and Tissue Repair

To characterize tissue‐level responses to Gel/aNEV treatment, transcriptomic profiling was performed on wound tissues. Differential expression analysis identified 119 differentially expressed genes (DEGs), including 48 upregulated and 71 downregulated genes in the Gel/aNEV group compared with Gel (Figure 11A). A focused heatmap of representative therapeutically relevant DEGs revealed a coordinated expression pattern, with reduced *Ccl28, Tlr4*, and *C5ar2* and increased *Mmp9, Adamts20, Wnt3a, Fn1, Hif1a*, and *Kdr* in Gel/aNEV‐treated wounds (Figure 11B). These changes were consistent with attenuation of inflammatory and complement‐associated signaling together with enhanced extracellular matrix remodeling, tissue regeneration, and angiogenesis.

**FIGURE 11:**
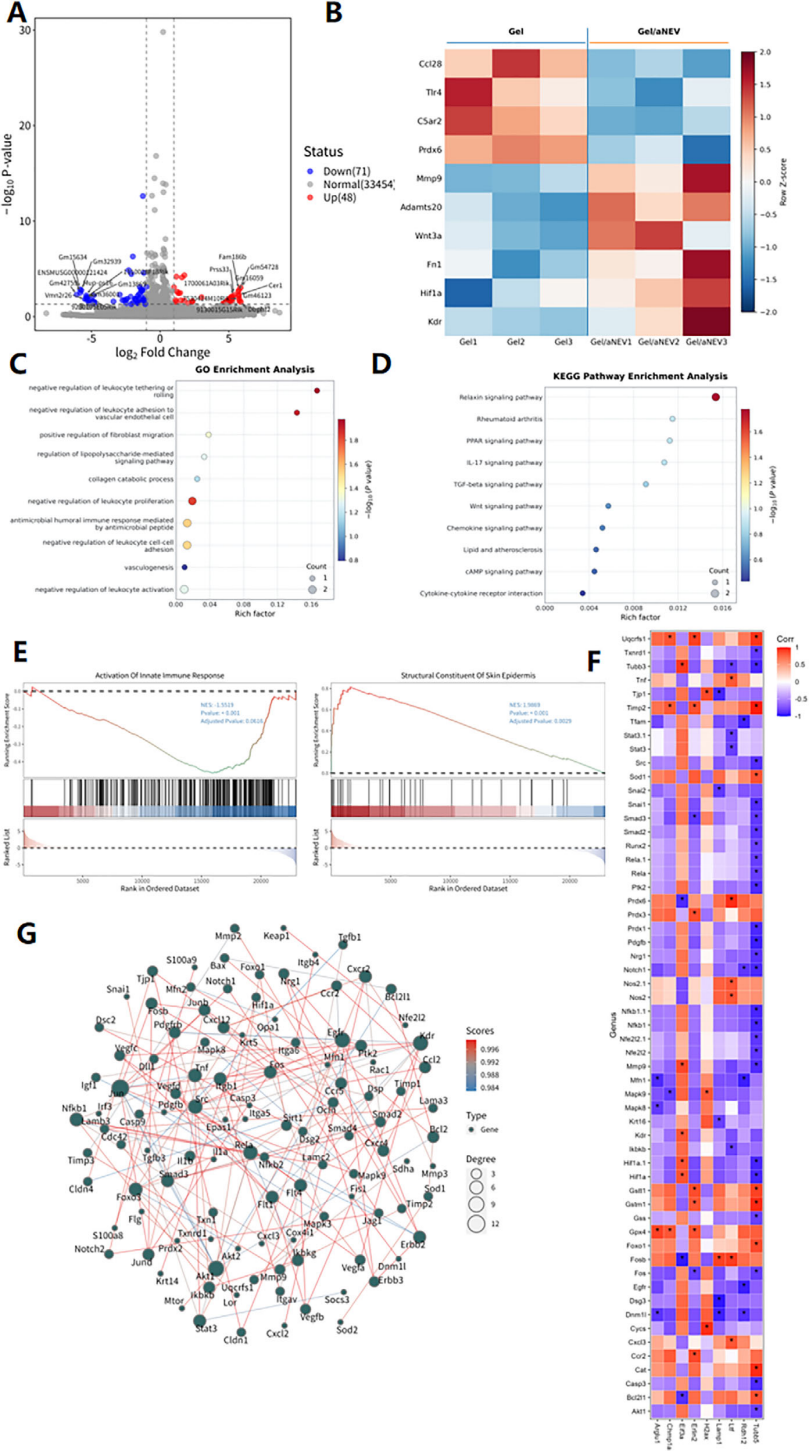
Transcriptomic profiling and multi‐omics integration of Gel/aNEV‐mediated wound repair. (A) Volcano plot showing differentially expressed genes between Gel/aNEV‐ and Gel‐treated wound tissues. Red and blue indicate upregulated and downregulated genes, respectively. (B) Heatmap of representative therapeutically relevant genes associated with inflammatory/complement regulation and tissue repair. Expression values are shown as row Z‐scores. (C) GO enrichment analysis highlighting biological processes related to immune regulation, leukocyte behavior, fibroblast migration, vascular responses, and extracellular matrix remodeling. Bubble size represents gene count, and color indicates enrichment significance. (D) KEGG pathway enrichment analysis showing representative immune‐ and repair‐associated pathways. Bubble size represents gene count and color indicates *p*‐value. (E) Representative GSEA plots showing negative enrichment of activation of innate immune response and positive enrichment of structural constituent of skin epidermis. NES and FDR values are indicated within each plot. (F) Correlation heatmap integrating differentially abundant aNEV proteins with wound‐tissue transcriptional responses; asterisks indicate statistically significant correlations. (G) Protein–protein interaction network of core wound‐response genes. Node size represents network degree and node color indicates network score. n = 3 per group. Visualization code for panels B–D was generated and refined with assistance from OpenAI Codex using the GPT‐5.6 Sol model. The plots were generated from the authors’ underlying transcriptomic data and statistical results, and all AI‐assisted outputs were reviewed and verified by the authors.

GO enrichment analysis highlighted biological processes associated with negative regulation of leukocyte activation and vascular adhesion, regulation of lipopolysaccharide‐mediated signaling, fibroblast migration, collagen remodeling, and vasculogenesis (Figure 11C). KEGG analysis further identified immune‐ and repair‐associated pathways including PPAR, IL‐17, TGF‐β, Wnt, chemokine, cAMP, and relaxin signaling among the top‐ranked pathways (Figure 11D). Representative GSEA provided complementary pathway‐level evidence: activation of innate immune response showed negative enrichment (NES = −1.55, FDR = 0.0616), whereas structural constituent of skin epidermis was positively enriched (NES = 1.99, FDR = 0.0029), supporting a shift from inflammatory activation toward epidermal repair (Figure 11E).

A broader therapeutically oriented GSEA analysis was additionally performed and is presented in Figure S7. GO‐GSEA showed significant negative enrichment of leukocyte‐mediated immunity (NES = −1.54, FDR = 0.00279), together with negative enrichment directions for innate immune and complement‐associated programs, whereas epidermis development, keratinocyte differentiation, and epidermal cell differentiation were positively enriched. KEGG‐GSEA similarly showed negative enrichment of several inflammatory pathways and complement/coagulation cascades, accompanied by positive enrichment of Wnt‐associated repair signaling (Figure S7).

Finally, transcriptome–proteome correlation analysis revealed coordinated relationships between aNEV‐associated proteins and wound‐tissue genes involved in inflammation, redox regulation, extracellular matrix remodeling, and regeneration (Figure 11F). Consistent with these cross‐omics associations, integrated transcriptomic–proteomic enrichment analysis further highlighted GO terms related to reactive oxygen species and oxidative stress responses, hypoxia, epithelial cell proliferation, antioxidant activity, and extracellular matrix‐associated functions, while KEGG analysis identified inflammatory and repair‐related pathways including TNF, PI3K–Akt, IL‐17, focal adhesion, and AGE–RAGE signaling in diabetic complications (Figure S8). PPI network analysis further demonstrated that these transcriptional responses formed an interconnected immune–repair network rather than isolated gene changes (Figure 11G). Together, these findings support coordinated tissue‐level reprogramming involving inflammatory regulation, oxidative stress control, extracellular matrix remodeling, and regenerative responses following Gel/aNEV treatment.

### 2.10 Multi‐Omics Integration Supports Concordance Between the scRNA‐seq‐Guided C2 Signature and Tissue‐Level Responses

To bridge the single‐cell discovery layer with tissue‐level transcriptional responses, we performed systematic multi‐omics integration using the atlas‐derived C2 signature as an analytical reference. CIBERSORTx analysis revealed no statistically significant differences in the estimated relative abundance of major cellular populations, including endothelial cells, fibroblasts, macrophages, and neutrophils, between the Gel/aNEV and Gel groups (Figure 12A). These results indicate that the observed therapeutic responses were not accompanied by detectable large‐scale shifts in the relative proportions of the major cell populations assessed. Consistent with this observation, UCell analysis showed that the transcriptional activity of the endogenous C2‐associated neutrophil program remained comparable following Gel/aNEV treatment (*p* = 0.70, Figure 12B). Together, these findings suggest that Gel/aNEV‐associated tissue responses occur without detectable broad changes in major cell‐type composition and are accompanied by transcriptional alterations within the wound microenvironment.

**FIGURE 12:**
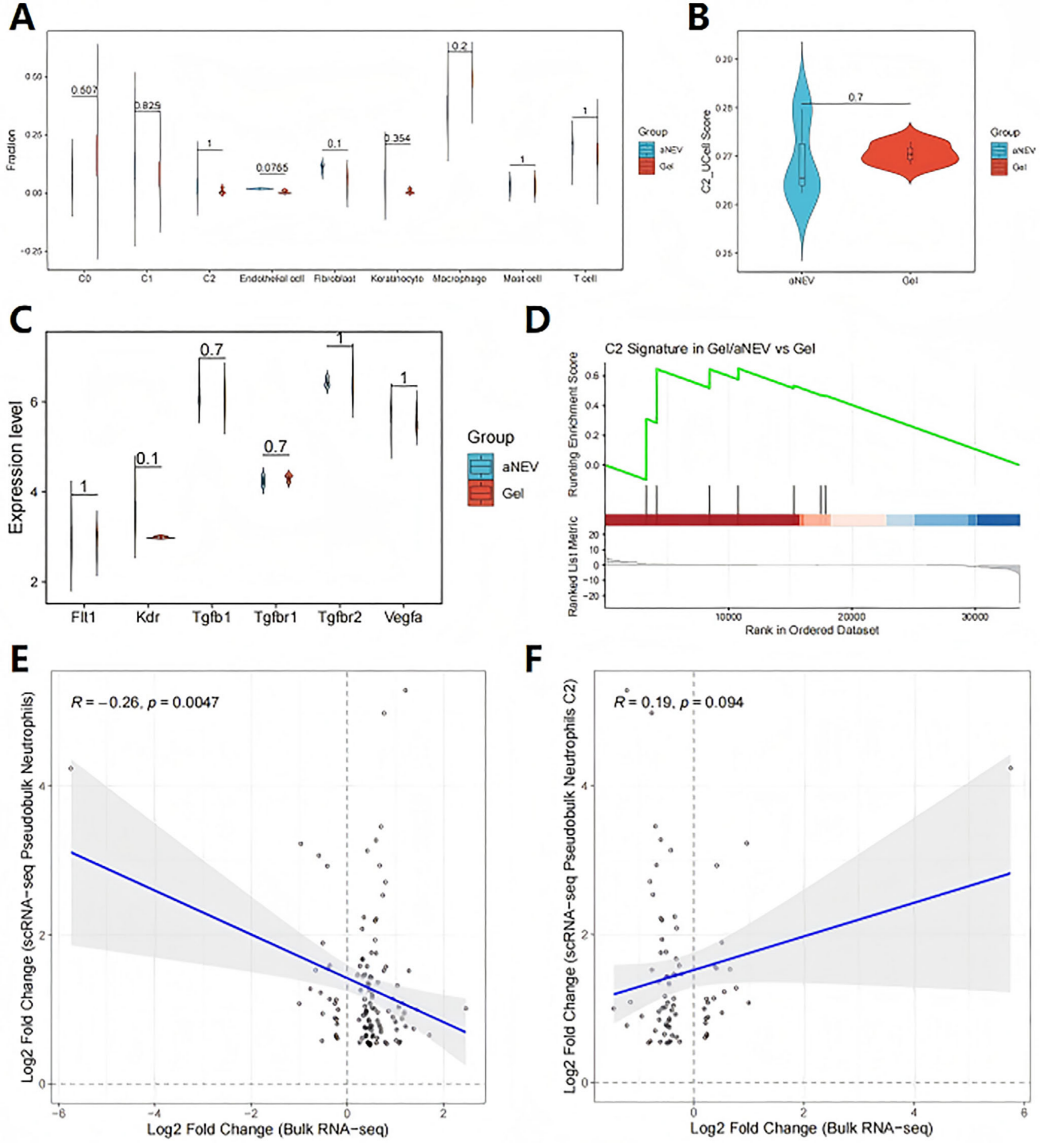
Multi‐omics integration comparing single‐cell‐derived signatures with bulk tissue transcriptional responses. (A) CIBERSORTx cellular deconvolution of bulk RNA‐seq (Gel/aNEV vs. Gel, n = 3) using the mouse diabetic wound scRNA‐seq reference atlas, showing relative proportions of endothelial cells, fibroblasts, macrophages, neutrophils, and other cell types. (B) UCell scoring of the endogenous C2‐associated neutrophil signature in bulk transcriptomes, showing comparable scores following Gel/aNEV treatment (*p* = 0.70, n = 3). (C) Cross‐scale comparison of CellChat‐predicted ligand–receptor pairs (Vegfa–Flt1/Kdr and Tgfb1–Tgfbr1/Tgfbr2) with bulk RNA‐seq changes, showing no significant differences in the selected signaling axes (*p* > 0.05). (D) Gene set enrichment analysis (GSEA) of the C2‐associated gene signature in the ranked bulk transcriptomic gene list, showing enrichment in Gel/aNEV‐treated wounds relative to Gel‐treated wounds (NES = 1.45, ^*^ *p* = 0.032). (E) Pseudobulk‐bulk correlation: scatter plot of neutrophil pseudobulk log_2_ fold changes vs. real bulk DEG log_2_ fold changes (Gel/aNEV vs. Gel), revealing a modest but significant negative correlation (Pearson R = −0.26, ^**^ *p* = 0.0047). (F) Correlation between C2 neutrophil pseudobulk and whole‐wound bulk log2 fold changes, showing a weak, non‐significant positive correlation (Pearson R = 0.19, *p* = 0.094). Data in (A–C) are displayed as violin plots with embedded box plots. GSEA in (D) was performed using the clusterProfiler R package. Correlation significance in (E) and (F) was determined by a two‐tailed Pearson test. ^*^ *p* < 0.05, ^**^ *p* < 0.01, ^***^ *p* < 0.001.

To assess the concordance between tissue‐level transcriptional responses and the atlas‐defined C2 state, a C2‐associated gene signature was derived from the scRNA‐seq analysis. GSEA revealed enrichment of this signature in Gel/aNEV‐treated wounds relative to Gel‐treated wounds (NES = 1.45, ^*^ *p* = 0.032; Figure 12D). These findings indicate cross‐scale concordance between the atlas‐derived C2‐associated signature and the transcriptional profile of Gel/aNEV‐treated wound tissue, without implying complete equivalence between the endogenous C2 state and the tissue‐level response.

Expression levels of canonical regenerative signaling axes, including Vegfa–Flt1/Kdr and Tgfb1–Tgfbr1/Tgfbr2, remained comparable between Gel/aNEV‐treated and Gel‐treated wounds (Figure 12C). Notably, improved macroscopic and histological healing outcomes were observed despite the absence of marked changes in these signaling axes. These findings indicate that the observed tissue‐repair phenotype was not accompanied by robust transcriptional upregulation of the selected canonical pro‐angiogenic or TGF‐β‐associated pathways and may instead coexist with the broader immunoregulatory and microenvironmental changes identified in the transcriptomic analyses.

To compare scRNA‐seq‐derived neutrophil transcriptional changes with tissue‐level responses, we aggregated neutrophil subsets into pseudobulk expression profiles and compared their fold‐change patterns with bulk wound‐tissue DEGs (Gel/aNEV vs. Gel). Neutrophil pseudobulk fold changes showed a modest but statistically significant negative correlation with bulk tissue‐level fold changes (Pearson R = −0.26, ^**^ *p* = 0.0047; Figure 12E). This inverse association indicates that the relationship between the neutrophil‐derived transcriptional reference and the whole‐wound response is not characterized by simple directional concordance. Given the distinct cellular scales represented by pseudobulk neutrophil and whole‐tissue transcriptomes, this relationship is best interpreted as a cross‐scale association rather than evidence of a direct regulatory cascade.

As an additional cross‐scale comparison, sample‐level pseudobulk expression profiles of the C2 neutrophil subcluster were compared with tissue‐level bulk transcriptional changes. A weak positive correlation was observed between C2 pseudobulk and whole‐tissue fold changes, although it did not reach statistical significance (Pearson R = 0.19, *p* = 0.094; Figure 12F). Accordingly, this analysis should be interpreted as directional rather than confirmatory evidence. Considered together with C2‐module enrichment and cellular deconvolution, these complementary analyses suggest partial cross‐scale concordance between the atlas‐derived neutrophil signature and the transcriptional landscape associated with Gel/aNEV‐treated wounds, while also highlighting the complexity of relating cell‐type‐specific signatures to whole‐tissue responses.

## 3 Discussion

The central advance of this study lies in translating a single‐cell‐derived neutrophil signature into an atlas‐guided extracellular vesicle therapeutic platform. Previous single‐cell studies have characterized neutrophil heterogeneity in diabetic wounds [67, 68], whereas our analysis further identified a CD55‐enriched C2 state with complement‐regulatory and reparative features and used this information to guide subsequent aNEV engineering. The rapid decline of endogenous CD55^+^ neutrophils during wound progression suggests that this regulatory program is transient and may become insufficient to counter persistent diabetic inflammation. This observation also extends previous work describing CD55‐positive neutrophil‐derived vesicles involved in inflammation resolution [13]. Importantly, targeted transcriptional validation showed that HG+LPS‐conditioned neutrophils partially recapitulated selected C2‐associated features, including increased expression of *Ltf*, *Chil3*, and *Camp* together with maintained high *Cd55* expression. Because *Jun* was also induced, the conditioned state is more appropriately considered C2‐like rather than transcriptionally equivalent to endogenous C2 neutrophils. These findings strengthen the experimental link between atlas‐derived marker selection, ex vivo neutrophil conditioning, and subsequent aNEV generation without implying complete phenotypic equivalence.

This atlas‐to‐engineering relationship also provides a biological basis for the “waste‐to‐treasure” concept. Rather than globally suppressing disease‐associated neutrophils, inflammatory and metabolic cues were repurposed ex vivo to enrich selected regulatory characteristics and generate a cell‐free therapeutic product. Compared with approaches using unstimulated neutrophils or single‐factor activation [17], HG+LPS conditioning generated aNEVs enriched in both complement‐associated and immunoregulatory components. Proteomic analysis identified lactoferrin, LAMP1, and Chmp1a among the altered cargos, distinguishing this profile from EV systems in which immunomodulation is predominantly mediated through nucleic‐acid or soluble cargos [18, 69]. In this context, “waste‐to‐treasure” represents the therapeutic repurposing of inflammatory‐cell plasticity: a pathological immune‐cell state is decoded and selectively redirected into a regenerative engineering strategy.

Platelet‐derived extracellular vesicles (pEVs) provide an important benchmark for blood‐derived EV therapy. Burnouf and colleagues have shown that pEVs promote angiogenesis, cell migration, inflammatory regulation, and diabetic wound repair and can be incorporated into gelatin‐based wound dressings [16, 17]. More recent work has highlighted the translational potential of pEV‐loaded hydrogel bandages, supported by established blood‐product sourcing and processing infrastructure [70]. The rationale for aNEVs is therefore not that they are universally superior to pEVs, but that they provide a distinct immunological configuration in which vesicular cargos coexist with membrane‐associated CD55. Consistent with this difference, Gel/aNEV promoted CD206‐associated macrophage polarization while reducing inflammatory macrophage features. Similar M1‐to‐M2 shifts have been reported with pEVs and MSC‐derived exosomes, including miR‐223/Pknox1‐ and PTEN/AKT‐associated mechanisms [12, 16, 71, 72]. Because EV source, dose, macrophage model, delivery material, and readouts differ substantially among studies, these findings are best interpreted as mechanistic complementarity rather than direct cross‐study potency ranking.

The expanded analyses are consistent with a multicomponent model involving both membrane‐associated and vesicular components of aNEVs. Membrane‐associated CD55 provides a plausible molecular basis for the observed complement‐associated effects, given its established role in accelerating the decay of C3/C5 convertases. In the present study, reduced C3a levels were accompanied by decreased *C5ar1*/*C5ar2* expression and increased *Cfh* expression, a pattern consistent with attenuation and rebalancing of complement‐associated signaling rather than indiscriminate suppression of complement function. Current DFU immunomodulatory approaches frequently target individual inflammatory mediators such as TNF‐α or IL‐1β [63, 73], whereas receptor‐specific complement therapies such as avacopan act at defined nodes [58, 59]. Localized aNEV delivery may therefore offer a means of modulating multiple complement‐associated readouts within the wound microenvironment, although the specific causal contribution of CD55 remains to be established by direct perturbation studies.

In parallel, the vesicular cargo profile provides a complementary macrophage‐regulatory dimension. Ltf was prominently enriched in HG+LPS‐derived aNEVs [54], while Gel/aNEV treatment was associated with increased macrophage *Lrp1* expression, reduced *Nlrp3* expression and Caspase‐1 levels, and increased *Il4*, *Stat6*, and *Pparg* expression. Lactoferrin can interact with LRP1 expressed by activated macrophages, and LRP1‐associated lactoferrin delivery has been linked to reduced inflammatory signaling and NLRP3‐associated responses [56]. The concurrent reduction in *Nlrp3* expression and Caspase‐1 levels is consistent with attenuation of an inflammasome‐associated inflammatory response [52], whereas STAT6‐ and PPARγ‐associated signaling has been implicated in macrophage inflammatory resolution and efferocytic activity [53]. Together with the reported immunoregulatory activities of neutrophil‐derived cargos such as itaconate [13, 57], these observations are consistent with a multicomponent model in which cargo‐associated signals may complement membrane‐associated complement regulation in shaping macrophage responses. Future CD55‐neutralization or knockdown studies, lactoferrin depletion or blockade, LRP1 inhibition, RNA‐cargo profiling, and combinatorial perturbation experiments will be required to quantitatively resolve the relative contribution and interaction of these components.

Biomaterial‐assisted delivery further addresses the limited local persistence of freely administered EVs [74]. The experimental analyses directly demonstrated PKH67‐labeled aNEVs within the Gel/aNEV matrix, gradual composite protein‐equivalent release, and measurable adhesion to porcine skin. The intrinsic proteolytic susceptibility of gelatin provides a basis for wound‐associated matrix remodeling [21]. Importantly, elevated MMP‐9 is a recognized feature of chronic and diabetic wound fluid and has been analytically validated in patient‐derived wound samples, including active MMP‐9 measurements by Hugo, Bouchiat and colleagues [44]. Accordingly, the markedly greater degradation observed in glucose‐ and MMP‐9‐containing DM‐SWF than in PBS supports the responsiveness of the gelatin matrix to a proteolytic wound environment, rather than attributing elevated MMP‐9 specifically to the hydrogel. The mild mTG‐mediated crosslinking conditions remain advantageous compared with harsher chemical crosslinking strategies [19, 20], while the resulting matrix retains skin‐compatible mechanical properties [37]. Its proteolytic degradation profile may also provide a useful intermediate between rapidly degrading fibrin‐based matrices and highly persistent synthetic polymer systems [75, 76].

The delivery formulation also provides a quantitative basis for evaluating the administered aNEV dose. Gel/aNEV contained 3 × 10^8^ particles mL^−1^, corresponding to approximately 6 × 10^7^ particles in each 200‐µL wound application. A single application was associated with improved wound healing over the 14‐day observation period. This represents an experimentally effective dose in the present model and provides a practical starting point for subsequent dose–response optimization. Published EV wound systems employ substantially different particle concentrations and dosing schedules; for example, a clinical‐grade pEV gelatin formulation has been reported at approximately 1 × 10^11^ particles mL^−1^[70], underscoring the importance of considering EV source, cargo potency, delivery matrix, and administration frequency together rather than particle number alone.

The Day‐14 NTA analysis provides evidence for persistence of an aNEV‐sized nanoparticle population within the degrading matrix under wound‐mimetic in vitro conditions. After 14 days in DM‐SWF, residual Gel/aNEV contained nanoparticles with a mean diameter of approximately 140 nm and a D50 of approximately 119 nm, closely overlapping the characteristic size range of freshly prepared aNEVs, whereas blank Gel predominantly produced smaller gelatin‐associated particles. However, particle size alone cannot establish that these residual nanoparticles represent intact or biologically active aNEVs, nor does this in vitro analysis establish comparable vesicle persistence in vivo. Previous studies using transglutaminase‐crosslinked protein hydrogels have similarly reported prolonged EV‐associated retention or gradual EV release over comparable time frames [41, 42]. Thus, the present findings support the capacity of the matrix to maintain an aNEV‐sized nanoparticle population during prolonged incubation under wound‐mimetic conditions and motivate future studies directly assessing late‐phase recovery, structural integrity, and complement‐regulatory or macrophage‐modulating activity of released vesicles.

At the tissue level, transcriptomic sequencing and multi‐omics integration identified coordinated changes in inflammatory, complement‐associated, antioxidant, and regenerative programs following Gel/aNEV treatment rather than changes confined to a single downstream pathway. In contrast to growth‐factor‐centered approaches that primarily enhance proliferative responses [77], Gel/aNEV treatment was associated with reduced inflammatory and complement‐related signatures together with enrichment of epithelial differentiation, antioxidant, and tissue‐remodeling programs. A notable strength of this study is the integration of multiple analytical layers to compare atlas‐derived discovery with tissue‐level therapeutic responses. Cellular deconvolution suggested limited changes in the estimated proportions of major cell populations, whereas C2‐signature analysis supported partial concordance between atlas‐derived signatures and tissue transcriptional responses. Ligand–receptor and pseudobulk‐to‐bulk analyses further revealed cross‐scale associations between neutrophil‐derived transcriptional references and whole‐tissue responses. Together, these analyses provide a cross‐scale analytical framework connecting single‐cell‐guided therapeutic design with tissue‐level responses to the biomaterial intervention.

Viewed in the context of existing therapeutic strategies, the present study advances the field at three interconnected levels. First, whereas Januszyk et al. and Dong et al. mapped neutrophil heterogeneity in diabetic wounds [67, 68], and Hsu et al. characterized large senescent neutrophil‐derived LAND‐Vs (∼1 µm) involved in inflammation resolution [13], our strategy uses an atlas‐defined neutrophil state to guide the generation of nanoscale aNEVs (120–140 nm) from experimentally controllable HG+LPS‐conditioned neutrophils. This provides a practical extension from descriptive immune‐cell heterogeneity to engineered vesicle therapeutics. Second, the mTG‐gelatin matrix enables aNEV incorporation under mild conditions and provides local retention, tissue adhesion, and protease‐sensitive remodeling. This delivery profile differs from hydrogel systems requiring crosslinking conditions that may be less favorable for sensitive biological cargos [22, 23], and from rapidly degrading matrices that may provide more limited retention [39]. Third, Gel/aNEV introduces a localized complement‐associated immunomodulatory strategy. In contrast to systemic complement inhibitors, which may require repeated administration and can increase susceptibility to infection [58, 61], local delivery of CD55‐enriched aNEVs was associated with modulation of complement‐related responses together with a shift toward reparative macrophage‐associated phenotypes, while potentially limiting systemic exposure. Together, these features position Gel/aNEV as an integrated atlas‐to‐vesicle‐to‐biomaterial strategy rather than an incremental modification of a single EV or hydrogel platform.

The complement‐regulatory interpretation is supported by convergent associations across multiple experimental levels. Nano‐flow cytometry demonstrated enrichment of surface CD55 on HG+LPS‐derived aNEVs; ELISA showed reduced C3a levels following Gel/aNEV treatment; in vivo analyses revealed coordinated changes in complement receptor expression and inflammatory mediators; and transcriptomic profiling identified attenuation of complement‐associated signatures. Together with the cargo‐associated macrophage responses described above, these findings are consistent with a cooperative multicomponent model in which membrane‐associated complement‐regulatory features and vesicular immunoregulatory signals are jointly associated with reduced inflammatory amplification and improved tissue‐repair responses. However, the relative causal contributions of CD55 and individual vesicular cargos cannot be resolved from the present data. Future studies incorporating CD55 neutralization or genetic silencing, lactoferrin depletion or blockade, LRP1 inhibition, and selective cargo perturbation will be required to define the contribution and interaction of these components.

Although integration of public scRNA‐seq datasets enabled cross‐study interrogation of diabetic wound heterogeneity, this approach has inherent limitations. Public datasets may differ in tissue collection, disease stage, sequencing depth, processing pipelines, batch structure, and available clinical metadata, and the relatively limited number of neutrophils recovered from the human DFU datasets restricted high‐resolution reconstruction of a human C2 counterpart. Moreover, cross‐species studies indicate that human and murine neutrophils share conserved core inflammatory transcriptional programs while retaining species‐specific features [78]. Therefore, the murine C2 state cannot be assumed to have a directly equivalent human counterpart and should be regarded as a mechanistic reference rather than a validated human‐equivalent population.

Future translational studies should therefore extend the atlas‐guided strategy in several directions. First, prospective acquisition of human DFU tissue and peripheral‐blood samples will enable higher‐resolution characterization of human neutrophil states and direct comparison with the murine C2 signature. Human‐derived aNEVs should subsequently be generated from multiple donors, including diabetic and non‐diabetic backgrounds, and evaluated for CD55 abundance, cargo composition, complement regulation, and macrophage‐modulating activity. In parallel, human iPSC‐derived neutrophils may provide a scalable and genetically tractable cell source for standardized aNEV production and mechanistic studies involving CD55 editing or cargo manipulation. Second, future manufacturing development should address neutrophil isolation reproducibility, donor variability, scalable EV purification, batch‐to‐batch consistency, storage, sterility, and mechanism‐linked potency assays. MISEV2023 and emerging clinical‐grade EV manufacturing frameworks provide relevant standards for EV characterization, source traceability, purity, stability, and quality control [79, 80].

A further clinically relevant dimension is diabetic peripheral neuropathy (DPN), which contributes to diabetic foot disease through sensory dysfunction, nerve‐fiber degeneration, Schwann‐cell abnormalities, and neuroinflammation. Cheng et al. recently highlighted the cell‐type‐specific nature of mTOR dysregulation in DPN, emphasizing that neuronal hyperexcitability and Schwann‐cell‐mediated regenerative responses may require distinct molecular considerations [81]. Related work from this group further underscores the importance of neural–immune–tissue communication in tissue homeostasis and regeneration [82]. These perspectives extend diabetic wound healing beyond an exclusively inflammatory or vascular disorder. Future studies incorporating neuropathy‐associated wound models, Schwann‐cell and sensory‐nerve readouts, and neuroimmune analyses may clarify whether the complement‐ and macrophage‐associated immunomodulatory responses observed with Gel/aNEV treatment are also accompanied by beneficial effects on the neural component of diabetic wound repair.

More broadly, effective diabetic wound therapy will likely require complementary rather than competing regenerative strategies. Platelet‐derived EVs provide strong trophic, pro‐migratory, angiogenic, and immunomodulatory activities [16, 17], whereas the present aNEV platform is distinguished by its capacity to couple complement‐associated regulation with macrophage reprogramming. In parallel, stem‐cell‐based and EV‐based cell‐free therapies, organic or inorganic biomaterials, ROS‐modulating systems, and emerging neuroregenerative approaches address additional dimensions of diabetic wound pathology [83, 84]. Integrating these complementary strengths may ultimately enable coordinated control of inflammation, oxidative stress, vascular regeneration, tissue reconstruction, and peripheral nerve repair, thereby facilitating collaborative translation toward more comprehensive diabetic foot therapies.

Overall, the findings establish an atlas‐guided immunoengineering framework in which a transient neutrophil regulatory program is identified, selected features of this program are partially recapitulated through a controllable ex vivo conditioning strategy, and the resulting aNEVs are delivered using a wound‐compatible biomaterial. In this context, the “waste‐to‐treasure” principle extends beyond simple neutrophil suppression by repurposing disease‐associated immune plasticity as a therapeutic design resource. Future integration of prospective human single‐cell atlases, donor‐specific molecular information, human‐ or iPSC‐derived aNEVs, and dose optimization may further extend this framework toward more individualized therapeutic development. Together, atlas‐derived target discovery, multicomponent membrane‐ and cargo‐associated immunoregulation, and localized biomaterial delivery provide a mechanistically informed strategy for modulating complement‐ and macrophage‐associated responses in chronic diabetic wounds.

## 4 Materials and Methods

### 4.1 Experimental Animals and Cell Sources

The animal experimentation was approved and supervised by the Animal Care and Use Committee of Shanghai Sixth People's Hospital (Animal Experiment Registration number: DWSY2025‐0017 and Animal Welfare Ethics acceptance number: DWLL2026‐0012).

Specific pathogen‐free (SPF) C57BL/6 mice (8–10 weeks old) were housed in the animal facility of Shanghai Sixth People's Hospital. Bone marrow neutrophils were isolated from mouse femurs and tibias. High‐purity neutrophils were obtained via Percoll density gradient centrifugation for subsequent experiments.

### 4.2 Public Database Information

Publicly available single‐cell RNA sequencing data included: human DFU datasets (GSM7908977, GSM7908978) and data from a diabetic wound model in db/db mice (C57BLKS/J background, GSM8013669) along with normal healthy control data (GSM7025081).

### 4.3 Isolation and Culture of Murine Bone Marrow Neutrophils

Bone marrow neutrophils were isolated from 8‐week‐old male C57BL/6 mice by Percoll density gradient centrifugation (52%, 62%, 76%). Briefly, femurs and tibias were flushed with Hanks’ balanced salt solution (HBSS) containing 15 mM EDTA and 0.5% BSA, layered onto Percoll gradients, and centrifuged at 1060 × g for 30 min. Cells at the 62%–76% interface were collected and purified using Histopaque‐1119. Purity (>90% Ly6G^+^) was confirmed by flow cytometry (BD FACSCanto II, FlowJo v10.8). For functional assays, isolated neutrophils were cultured in RPMI 1640/20% FBS supplemented with the CLON‐G survival cocktail (Q‐VD‐Oph, DFO, Hsp70, NAC, Nec‐1s, G‐CSF) and treated with high glucose (HG, 35 mM), LPS (100 ng mL^−1^), or HG+LPS for 24–48 h [13, 85]. CD55 expression under various stimulatory conditions was analyzed by flow cytometry using a PE‐conjugated anti‐Ly6G antibody and an anti‐CD55 antibody.

To assess the extent to which HG+LPS conditioning recapitulated selected molecular features of the atlas‐defined C2 neutrophil state, cells were additionally collected after 24 h for targeted RT‐qPCR analysis of atlas‐derived neutrophil‐state markers. The C2‐associated genes *Cd55*, *Ltf*, *Chil3*, and *Camp* were analyzed together with the C0‐ and C1‐associated comparator genes *Csf3* and *Jun*, respectively. Detailed experimental procedures are provided in the Supplementary Methods.

### 4.4 Isolation, Characterization, and Proteomic Profiling of Neutrophil‐Derived Extracellular Vesicles (NEVs)

Neutrophil‐conditioned culture medium was centrifuged at 1500 × *g* for 5 min. The resulting supernatant was collected and centrifuged at 3000 × *g* for 20 min, and the pellet was recovered as the NEV preparation [13]. Physical properties were assessed by nanoparticle tracking analysis (ZetaView PMX 110) and transmission electron microscopy (negative staining with 2% uranyl acetate). Protein content was quantified by BCA assay. EV markers (TSG101, Alix, CD81) and neutrophil‐specific markers (Ly6G, CD55) were detected by Western blot [13, 86]. Following protein transfer, PVDF membranes were physically sectioned according to the expected molecular‐weight ranges before primary‐antibody incubation, while retaining the corresponding molecular‐weight marker lane for orientation. Individual membrane sections were subsequently probed with the indicated antibodies (Figure S9). CD55 surface expression was further quantified by nano‐flow cytometry (NanoFCM). For functional studies, PKH67‐labeled NEVs were co‐cultured with RAW 264.7 macrophages, and the internalization process was subsequently visualized by confocal microscopy. For proteomic analysis, NEVs from HG+LPS‐stimulated (aNEVs) and control neutrophils were subjected to direct DIA quantitative proteomics: proteins were extracted, digested with trypsin, and analyzed by nano‐flow LC‐MS/MS on a Vanquish Neo UHPLC system coupled to an Orbitrap Astral mass spectrometer equipped with an Easy‐spray (ESI) ion source. Database searching and protein quantification were performed using DIA‐NN against the *Mus musculus* UniProt database. Differentially abundant proteins were identified using Student's *t*‐test, with thresholds of fold change ≥ 1.5 or ≤ 0.67 and *p* < 0.05. Fold changes were calculated as the abundance ratio of HG+LPS‐derived aNEVs to control‐derived NEVs. Detailed protocols for extracellular vesicle isolation, nanoparticle tracking analysis, transmission electron microscopy, nano‐flow cytometry, and Western blot characterization are provided in the Supporting Information.

### 4.5 Preparation of Injectable Gelatin Hydrogel and Gel/aNEV Composite

Type A gelatin was dissolved in distilled water above 37°C with vortexing to prepare a 4% (w v^−1^) pre‐gel solution. Microbial transglutaminase (mTG, 0.1 g L^−1^ in PBS) was added at 40 µL per mL pre‐gel, and the mixture was stirred at 37°C until homogeneous, undergoing slow, irreversible cross‐linking within 5 min to form a translucent, non‐flowing gel. For Gel/aNEV composite preparation, aNEV stock solution (3 mg mL^−1^ protein, 5 × 10^9^ particles mL^−1^) was incorporated into the pre‐gel solution before mTG addition to achieve final concentrations of 180 µg mL^−1^ aNEV protein, 3 × 10^8^ particles mL^−1^.

### 4.6 Preparation of Diabetic Microenvironment–Mimetic Simulated Wound Fluid

Diabetic microenvironment–mimetic simulated wound fluid (DM‐SWF) was prepared using commercially available artificial wound fluid exudate containing electrolytes and bovine serum albumin (Mimic Fluid, China; Cat. No. MSB02700) as the basal medium. D‐Glucose was added to a final concentration of 25 mM, and active MMP‐9 was added immediately before use to a final concentration of 200 ng mL^−1^. The supplemented medium was gently mixed, maintained at pH 7.4 ± 0.1, and used for hydrogel swelling and degradation assays at 37°C.

### 4.7 Confocal Visualization of aNEV Incorporation Into Gel/aNEV

To directly verify successful incorporation of aNEVs into the gelatin matrix, aNEVs were labeled with the fluorescent membrane dye PKH67 before hydrogel preparation. PKH67‐labeled aNEVs were incorporated into the gelatin pre‐gel prior to mTG‐mediated crosslinking. After gel formation, Gel/aNEV samples were examined using a Leica confocal laser scanning microscope. Blank Gel prepared under identical conditions served as the negative control. The presence and spatial distribution of PKH67‐positive fluorescence within the crosslinked hydrogel matrix were used as direct evidence of successful aNEV encapsulation. Representative confocal images are provided in Figure S6A.

### 4.8 Characterization of Gel/aNEV

Hydrogel samples were synthesized as described, photographed, and then lyophilized. The lyophilized samples were sectioned with a blade, sputter‐coated with gold, and observed using scanning electron microscopy (SEM, Phenom XL) to examine pore size distribution. The average pore diameter was calculated from measurements of ≥ 60 randomly selected pores using ImageJ software.

The porosity of the lyophilized hydrogels was determined. Briefly, a lyophilized sample (volume: 400 µL) was weighed (recorded as m_0_). It was then immersed in absolute ethanol, removed after a period, gently blotted to remove excess surface ethanol, and weighed again (m₁). Porosity was calculated using the following formula (n = 3):

$$porosity\;\left(\%\right)=\frac{m_{1}-m_{0}}{\rho \times V}\times 100$$

where ρ is the density of ethanol, and V is the volume of the hydrogel.

A newly synthesized hydrogel sample was weighed (w_0_). It was then immersed in PBS or DM‐SWF. At predetermined time points, the hydrogel was removed, blotted with wet filter paper to remove excess surface PBS or DM‐SWF, and weighed (w_t_). Swelling continued until equilibrium was reached. The swelling ratio was calculated as follows (n = 3):

$$Swelling\;Ratio\;\left(\%\right)=\frac{w_{t}-w_{0}}{w_{0}}\times 100$$

For water absorption, a lyophilized hydrogel was weighed (m₀). It was immersed in deionized water, removed at set intervals, blotted, and weighed (m_t_). The water absorption ratio and the maximum equilibrium water absorption ratio (EWR) were calculated (n = 3):

$$Water\;Absorption\;Ratio\;\left(\%\right)=\frac{m_{t}-m_{0}}{m_{0}}\times 100$$

Sterilized hydrogels were incubated in PBS or DM‐SWF at 37°C with shaking. At predetermined time points, samples were removed, rinsed with deionized water to remove residual PBS or DM‐SWF, lyophilized, and weighed (m_d_). At the start, three random hydrogel samples were lyophilized and weighed (m₀) as the initial dry weight. The degradation rate was calculated as follows (n = 3):

$$Degradation\;Rate\;\left(\%\right)=\frac{m_{0}\;-\;m_{d}}{m_{0}}\times 100$$

Gel and Gel/aNEV specimens were immersed in 2 mL of sterile PBS and incubated at 37°C under static conditions. At 6 h, 12 h, and days 1, 3, and 7, 100 µL of release medium was collected and replaced with an equal volume of fresh PBS. Protein‐equivalent concentrations were determined using a BCA assay after reagent‐blank and 0‐h baseline correction. Relative protein‐equivalent release was calculated as follows (n = 3):

$$R_{t}\left(\%\right)=\frac{C_{t}}{C_{0}}\times 100$$

where *C_t_* is the corrected protein‐equivalent concentration in the release medium at time *t*, and *C*_0_ is the corrected initial reference concentration of the corresponding specimen. Three independent specimens were analyzed per group, with technical triplicate measurements.

Hydrogel samples (10 mm diameter, 4 mm height) were subjected to uniaxial compression testing using a universal testing machine (Instron 5567, 50 N load cell) at a compression rate of 5 mm min^−1^. The maximum compressive stress and failure strain were recorded. The compressive modulus was calculated from the slope of the linear region corresponding to the initial 0%–5% strain (n = 4).

The tissue adhesive properties of Gel and Gel/aNEV were evaluated using an ex vivo porcine‐skin lap‐shear assay. Porcine skin was cut into rectangular strips measuring 5.0 × 1.5 cm. Two skin specimens were overlapped over a 2.5 × 1.5 cm region, corresponding to an effective adhesion area of 3.75 cm^2^. Gel or Gel/aNEV was introduced between the opposing skin surfaces within the overlapping region, followed by mTG‐mediated crosslinking at 37°C for 5 min. The bonded specimens were mounted on an Instron 5567 universal testing machine and separated at a constant displacement rate of 5 mm min^−^^1^ until interfacial failure occurred. The maximum detachment force (*F_max_*) was recorded, and adhesion strength was calculated as:

$$Tissue\;adhesion\;strength\;\left(\frac{N}{cm^{2}}\right)=\frac{F_{max}}{A}$$

where *A* represents the effective overlapping area (3.75 cm^2^). Six independent samples were tested for each group (n = 6).

### 4.9 Biocompatibility Assay

The cytocompatibility of the Gel/aNEV hydrogel was evaluated using human umbilical vein endothelial cells (HUVECs). Cells were seeded in 96‐well plates and co‐cultured with the following groups: Control (no treatment), Gel (empty hydrogel), and Gel/aNEV. The hydrogels were sterilized, and their extraction media were used for the assay. After 1 and 3 days of culture, cell viability was assessed using the CCK‐8 assay. Metabolic activity was quantified by measuring the optical density at 450 nm (OD_4__5_₀). Additionally, cells were stained using a Calcein/PI Cell Viability/Cytotoxicity Assay Kit (Beyotime Biotechnology, Shanghai, China). Images were captured using an inverted fluorescence microscope (Keyence BZX810, Osaka, Japan) to count live and dead cells (n = 3). Cell viability was calculated using the following formula:

$$Cell\;viability\;\left(\%\right)=\frac{Viable\;cells}{Total\;cells}\times 100$$

### 4.10 Scratch Wound Healing Assay

HUVECs were seeded into 6‐well plates and subjected to serum starvation overnight in serum‐free medium. Upon reaching confluence, a linear scratch was created using a 200 µL pipette tip. Cells were then treated with the following conditioned media: Control, LPS (100 ng mL^−1^), LPS + Gel, and LPS + Gel/aNEV, with fetal bovine serum (FBS) concentration maintained below 3%. Microscopic images were acquired at 0, 24, and 48 h, and the wound healing rate was quantified using ImageJ software to evaluate the pro‐migratory effect of aNEV. The cell migration rate was calculated as follows:

$$Healing\;rate\;\left(\%\right)=\left(1-\frac{new\;scratch\;width}{original\;scratch\;width}\right)\times 100$$

### 4.11 Macrophage Polarization Assay

RAW 264.7 macrophages were used to evaluate the immunomodulatory effects of Gel/aNEV. Cells were assigned to four experimental groups: Control, LPS (100 ng mL^−1^), LPS + Gel, and LPS + Gel/aNEV. After 24 h of treatment, macrophage phenotypes were characterized by flow cytometry using CD86 and CD206 as representative M1‐ and M2‐associated surface markers, respectively. Immunofluorescence staining was additionally performed using iNOS together with the pan‐macrophage marker CD68 to assess inflammatory macrophage activation, whereas CD206/CD68 co‐staining was used to visualize the reparative macrophage phenotype.

To expand the molecular characterization of macrophage immunomodulation, total RNA was extracted after treatment and reverse‐transcribed into cDNA. RT‐qPCR was performed to quantify the expression of the inflammatory genes *Ccr7, Il6*, and *Nlrp3*, together with the pro‐resolving/reparative macrophage‐associated genes *Il4, Pparg, Stat6*, and *Lrp1*. *Gapdh* served as the internal reference, and relative expression was calculated using the 2^−ΔΔCt^ method.

In parallel, culture supernatants were collected for quantification of Caspase‐1 by ELISA according to the manufacturer's instructions. These complementary analyses were used to assess both suppression of inflammatory activation and induction of a reparative macrophage program. Three independent biological replicates were analyzed for each condition.

RT‐qPCR primers were synthesized by Servicebio (Wuhan, China) or BioTNT Co., Ltd. (Shanghai, China), and the corresponding sequences are listed in Table S1.

### 4.12 Complement Assay

To evaluate the regulatory effects of aNEVs on complement‐associated responses under LPS stimulation, RAW 264.7 macrophages were randomly assigned to four groups: Control (standard culture), LPS (100 ng mL^−1^), LPS + Gel, and LPS + Gel/aNEV. Cells and culture supernatants were collected after 12 h under the indicated treatment conditions.

For transcriptional analysis, total RNA was isolated from treated macrophages and subjected to RT‐qPCR to quantify the expression of the complement‐associated genes *C5ar1, C5ar2, Cfh*, and *Itgam*, together with the inflammatory gene *Il1b*. *Cfh* encodes complement factor H, a major endogenous negative regulator of complement activation, whereas *Itgam* encodes the CD11b subunit of complement receptor 3 (CR3), thereby providing a broader assessment of complement activation, endogenous regulation, and complement‐associated cellular responses. *Gapdh* was used as the reference gene, and relative expression was calculated using the 2^−ΔΔCt^ method. Culture supernatants were analyzed by ELISA to quantify C3a as a functional readout of complement activation and IL‐6 as an inflammatory cytokine readout.

Intracellular oxidative stress was independently evaluated using the DCFH‐DA fluorescent probe. Following treatment, cells were incubated with DCFH‐DA, washed, and examined by fluorescence microscopy. Relative ROS generation was quantified from fluorescence intensity and normalized to the Control group. Three independent replicates (n = 3) were analyzed for each condition.

To evaluate the in vivo modulatory effects of Gel/aNEV on complement activation and the local inflammatory milieu, full‐thickness wound specimens encompassing the wound bed and adjacent tissue were harvested at postoperative day (POD) 7, rapidly frozen in liquid nitrogen, and stored at −80°C. For protein quantification, frozen tissues were pulverized under liquid nitrogen and homogenized in ice‐cold RIPA lysis buffer supplemented with protease and phosphatase inhibitor cocktails. The homogenates were centrifuged at 12 000 × g for 15 min at 4°C, and the resulting supernatants were quantified for C3a and IL‐6 using commercial ELISA kits (Elabscience, Wuhan, China) according to the manufacturer's protocols. Tissue lysates were adjusted to the same total protein concentration, as determined by BCA assay, prior to ELISA.

For transcriptional analysis, total RNA was extracted from homogenized wound tissues using the EZ‐press RNA Purification Kit (EZBioscience, Roseville, MN, USA) and reverse‐transcribed into cDNA using a Color Reverse Transcription Kit (EZBioscience). RT‐qPCR was subsequently performed with 2 × Color SYBR Green qPCR Master Mix (EZBioscience) and gene‐specific primers for *C3ar1*, *C5ar1*, and *C5ar2* (Table S1). *Gapdh* served as the internal reference, and relative expression levels were calculated using the 2^−ΔΔCt^ method.

To visualize the spatial distribution of the complement regulatory molecule CD55 alongside neutrophil infiltration within wound beds, immunofluorescence co‐staining for CD55 and Ly6G was performed on POD 7 paraffin‐embedded sections following the protocols detailed in the Histological and Immunofluorescence Analyses section. Three independent replicates (n = 3) were analyzed for each condition.

### 4.13 Diabetic Wound Model Establishment

Eight‐week‐old male C57BL/6 mice were fasted for 12 h with free access to water prior to streptozotocin (STZ) administration. STZ was freshly dissolved in sterile citric acid–sodium citrate buffer (pH 4.5) immediately before use. Diabetes was induced via a single intraperitoneal injection of STZ at 150 mg kg^−1^. Five days post‐injection, tail vein blood glucose levels were measured using a portable glucometer. Mice with sustained fasting blood glucose concentrations ≥ 16.7 mmol L^−1^ were considered successful diabetic models and included in the study. Prior to wound creation, anesthesia was induced and maintained with pentobarbital sodium. Two full‐thickness excisional skin wounds (8 mm in diameter) were created on the dorsal surface of each qualified mouse. Animals were randomly assigned to three treatment groups (n = 10 animals per group): Control (wounds receiving no treatment), Gel (wounds treated with blank gelatin hydrogel immediately after injury), and Gel/aNEV (wounds treated with the aNEV‐loaded composite hydrogel immediately after injury). Immediately after wound creation, 200 µL of Gel or Gel/aNEV was applied to each wound according to the assigned treatment group. Wound healing was monitored until postoperative day (POD) 14.

### 4.14 Wound Healing Assessment

Wounds were photographed using a digital camera on POD 0, 3, 7, 10, and 14. Wound areas were quantified using ImageJ software, and the wound healing rate was calculated accordingly. On POD 3, 7, and 14, subsets of mice from each group were randomly euthanized. Full‐thickness skin samples encompassing the wound center and surrounding tissue were harvested. A portion of each sample was immediately fixed in 4% paraformaldehyde for subsequent histological analysis. Additionally, samples collected on POD 7 were rapidly frozen in liquid nitrogen and stored at −80°C for transcriptomic (RNA‐seq) and molecular biological (qPCR, ELISA) analyses.

*Mmp9* expression was assessed by RT‐qPCR in POD 7 wound tissues from the Gel and Gel/aNEV groups as a tissue‐remodeling marker. Total RNA was extracted and reverse‐transcribed into cDNA, followed by RT‐qPCR using *Mmp9*‐specific primers (Table S1). *Gapdh* served as the internal reference gene, and relative expression was calculated using the 2^−ΔΔCt^ method.

### 4.15 Histological and Immunofluorescence Analyses

Fixed tissue samples were paraffin‐embedded, sectioned, and subjected to histological and immunostaining protocols. Hematoxylin and eosin (H&E) staining evaluated general tissue morphology and inflammatory cell infiltration, while Masson's trichrome staining assessed collagen fiber deposition and organization. Immunohistochemistry (IHC) was performed on POD 3 and 7 sections to detect pro‐inflammatory (IL‐1β, TNF‐α) and anti‐inflammatory (IL‐10) cytokines, with semi‐quantitative analysis by mean optical density. Immunofluorescence (IF) staining on POD 3 and POD 7 sections employed CD86 (M1 macrophages), CD206 (M2 macrophages), Ly6G (neutrophils), and CD55 (complement regulation) to characterize wound immune microenvironment composition, whereas POD 7 and POD 14 sections were stained for CD31/α‐SMA labeling for angiogenesis and granulation tissue evaluation.

### 4.16 Transcriptomic Sequencing (RNA‐seq) Analysis

Non‐strand‐specific mRNA libraries prepared from postoperative day 7 wound tissues were sequenced using a paired‐end 150‐bp strategy on the DNBSEQ‐T7RS platform. Raw reads were processed using fastp v0.23.2 and aligned to the Mus musculus GRCm39 reference genome with M31 annotation (Ensembl release 108) using HISAT2 v2.2.1. Gene‐level expression was quantified using StringTie v2.2.1, and differential expression analysis was performed using DESeq2 v1.42.1. Following quality control, alignment, and quantification of raw data, differentially expressed genes (DEGs) were identified using the criteria of | log_2_ (fold change)| ≥ 1 and false discovery rate (FDR) < 0.05. Gene Ontology (GO) enrichment analysis, Kyoto Encyclopedia of Genes and Genomes (KEGG) pathway analysis, and gene set enrichment analysis (GSEA) were performed, with particular attention to pathways related to inflammatory responses, complement activation, angiogenesis, and tissue remodeling.

### 4.17 Multi‐Omics Integration and Single‐Cell‐to‐Bulk Bridging Analysis

Integrative analyses were performed in R version 4.3.1 to relate single‐cell‐derived neutrophil signatures to bulk transcriptomic responses in Gel/aNEV‐ and Gel‐treated wounds (*n* = 3 biological replicates per group). CIBERSORTx was used to estimate cell‐type proportions using the mouse diabetic wound scRNA‐seq atlas as a reference. UCell was applied to quantify C2‐associated signature scores in individual bulk RNA‐seq samples. CellChat‐predicted ligand–receptor pairs, including VEGFA–FLT1/KDR and TGFB1–TGFBR1/TGFBR2, were examined alongside the expression of their corresponding genes in bulk RNA‐seq data. Neutrophil pseudobulk expression profiles were generated from the scRNA‐seq data, and Pearson correlation analysis was used to compare pseudobulk and whole‐wound bulk log_2_ fold changes for overlapping genes. Gene set enrichment analysis (GSEA) of the scRNA‐seq‐derived C2‐associated gene signature was performed using the clusterProfiler R package. Visualizations were generated using ggplot2 and ComplexHeatmap.

### 4.18 Statistical Analysis

Unless otherwise specified, *n* denotes the number of independent biological replicates included in the statistical analysis, and the exact sample size is provided in the corresponding figure legends. For in vitro experiments, biological replicates were derived from independently performed experiments, with at least three independent experiments included in the statistical analysis. For in vivo experiments, animals were randomly assigned to the indicated treatment groups (10 mice per group overall), and the number of animals or independent tissue samples included in each specific analysis is reported in the corresponding figure legend. For histological and immunofluorescence analyses, multiple non‐overlapping microscopic fields obtained from the same tissue specimen were averaged to generate a single biological replicate and were not treated as independent measurements. Similarly, technical measurements obtained from the same biological sample were averaged before statistical analysis. For bulk RNA‐seq, three independent wound‐tissue samples per group were analyzed unless otherwise indicated.

Comparisons between two groups were performed using an unpaired two‐tailed t‐test, with Welch's correction applied to the adhesion‐strength analysis. For comparisons involving more than two groups, one‐way analysis of variance (ANOVA) was followed by Tukey's test for pairwise comparisons or Dunnett's test for comparisons with a designated reference group, as appropriate. Two‐way ANOVA with appropriate multiple comparisons was applied to experiments involving two independent factors, such as treatment and time. Statistical analyses and graph generation were performed using GraphPad Prism 10.6 (GraphPad Software, San Diego, CA, USA).

Public single‐cell RNA‐seq and cell–cell communication analyses were conducted using Seurat, SingleR, CellPhoneDB, and CellChat. Cross‐scale integration of single‐cell‐derived signatures with bulk tissue transcriptional responses was performed in R version 4.3.1 using CIBERSORTx for cellular deconvolution and UCell for C2‐associated signature scoring. GSEA was performed using the clusterProfiler R package. Pearson correlation analysis was used to assess the relationship between neutrophil pseudobulk and whole‐wound bulk log_2_ fold changes for overlapping genes. Visualizations were generated using ggplot2 and ComplexHeatmap.

Statistical significance was defined as ^*^ *p* < 0.05, ^**^ *p* < 0.01, and ^***^ *p* < 0.001.

## 5 Conclusion

In conclusion, this study presents an atlas‐guided cell‐free immunomodulatory framework for diabetic wound repair. Single‐cell analysis identified a CD55‐enriched neutrophil state with complement‐regulatory and reparative‐associated features, which subsequently informed the ex vivo conditioning strategy used for aNEV generation. HG + LPS conditioning partially recapitulated selected C2‐associated molecular features and generated aNEVs enriched in membrane‐associated CD55 and immunoregulatory vesicular components. Incorporation of aNEVs into an mTG‐crosslinked gelatin hydrogel enabled localized delivery, measurable tissue adhesion, and protease‐sensitive matrix remodeling. Across in vitro and in vivo models, Gel/aNEV treatment was associated with reduced complement‐associated inflammatory responses, a shift toward reparative macrophage‐associated phenotypes, and improved diabetic wound‐healing outcomes. Transcriptomic and multi‐omics analyses further identified coordinated changes in inflammatory, redox, extracellular‐matrix, and regenerative programs and supported partial cross‐scale concordance with the atlas‐derived C2 signature. Collectively, these findings support a multicomponent model involving membrane‐associated complement regulation and cargo‐associated immunomodulatory signals, while direct perturbation studies will be required to define the causal contributions of CD55 and individual vesicular components. Further validation using human‐derived aNEVs, dose‐ranging studies, and standardized manufacturing and quality‐control procedures will be necessary to determine the translational potential of this platform.

## Author Contributions

Tairong Cui: Conceptualization, Methodology, Investigation, Writing – original draft. Jieying Li: Validation, Investigation. Jibing He: Methodology, Investigation, Formal analysis. Jia Wang: Methodology, Investigation, Data curation. Yun Qian: Methodology, Investigation. Yilin Jin: Methodology, Investigation. Liuqing Yang: Validation, Investigation. Zaijin Tao: Writing – review and editing, Methodology, Data curation, Conceptualization. Ziyun Li: Writing – review and editing, Methodology. Zhengsheng Chen: Methodology, Formal analysis. Wenjing Wang: Data curation. Jinglei Wu: Supervision, Resources, Funding acquisition, Writing – review and editing, Conceptualization. Xin Ma: Supervision, Resources, Funding acquisition, Writing – review and editing, Project administration, Conceptualization. All authors reviewed and approved the final manuscript.

## Ethics Statement

This retrospective observational study using archived clinical specimens was conducted in accordance with the Declaration of Helsinki and was approved by the Ethics Committee of Shanghai Sixth People's Hospital (Approval No: 2025‐KY‐195(K); Date of Approval: June 6, 2025). Written informed consent for the research use of archived clinical specimens and associated anonymized data and images was obtained from all included participants in accordance with the approved protocol. Archived full‐thickness dorsal foot skin specimens (∼1.0 cm × 1.0 cm × 0.3 cm) were retrospectively identified from patients with type 2 diabetes mellitus undergoing amputation surgery for diabetic foot ulcers. Research‐related retrieval, processing, and analysis of the archived specimens were conducted within the scope of the approved protocol. Patient sex was obtained from the clinical records. No identifying personal information—including patient names, initials, hospital registration numbers, dates of birth, social security numbers, or any other personal or identifying details—was used in this publication or any supplementary materials. All patient‐related images (hematoxylin–eosin staining, Masson's trichrome staining, and immunofluorescence images) were fully anonymized prior to inclusion. Written consents and permissions for the use of anonymized tissue specimens and images have been retained by the corresponding author and are available for review upon reasonable request by the journal in exceptional circumstances.

## Conflicts of Interest

The authors declare no conflicts of interest.

## Supporting information




**Supporting File**: advs78320‐sup‐0001‐SuppMat.docx.

## Data Availability

The bulk RNA‐seq data generated in this study have been deposited in the Gene Expression Omnibus (GEO) under accession number GSE348146. The publicly available scRNA‐seq datasets analyzed in this study are available under GEO accession numbers GSM7908977, GSM7908978, GSM8013669, and GSM7025081. Additional data supporting the findings of this study are available from the corresponding author upon reasonable request.
