# Supplementary material for: Atlas‐Guided CD55‐Enriched Neutrophil Extracellular Vesicles for Complement–Macrophage Modulation in Diabetic Wounds
Source: Adv Sci (Weinh). 2026 Oct 15:e78320. Online ahead of print. doi: 10.1002/advs.78320 (PMC13672684; doi:10.1002/advs.78320)
Supplement: Supplementary file 1 [file ADVS-9999-e78320-s001.docx]

Supporting Information

Atlas-Guided CD55-Enriched Neutrophil Extracellular Vesicles for Complement–Macrophage Modulation in Diabetic Wounds

*Tairong Cui ^1#^, Jieying Li ^2#^, Jibing He* *^1#^, Jia Wang ^1#^, Yun Qian ^1#^, Yilin Jin ^3^, Liuqing Yang ^1^, Zaijin Tao ^1^, Ziyun Li ^1^, Zhengsheng Chen ^1^, Wenjing Wang ^4^, Xin Ma ^1^*, Jinglei Wu ^2^***

*^1^ Department of Orthopedic Surgery, Shanghai Sixth People’s Hospital Affiliated to Shanghai Jiao Tong University School of Medicine, Shanghai 200233, China.*

*^2^ Shanghai Engineering Research Center of Nano-Biomaterials and Regenerative Medicine, College of Biological Science and Medical Engineering, Donghua University, Shanghai 201620, P. R. China*

*^3^ Fudan University School of Nursing, Shanghai, 200030, China.*

*^4^ Department of Clinical Laboratory, Shanghai Sixth People’s Hospital Affiliated to Shanghai Jiao Tong University School of Medicine, Shanghai 200233, China.*

*^#^ These authors contributed equally to this manuscript.*

** Correspondence: Xin Ma (*[*maxin@sjtu.edu.cn*](mailto:maxin@sjtu.edu.cn)*) and Jinglei Wu (*[*jw@dhu.edu.cn*](mailto:jw@dhu.edu.cn)*)*

Contents Overview

To improve accessibility and reproducibility, the supplementary information is organized into the following sections:

| Section | Content |
| --- | --- |
| Supplementary Methods S1 | Major Reagents and Instruments |
| Supplementary Methods S2 | Neutrophil isolation and culture |
| Supplementary Methods S3 | EV isolation and characterization |
| Supplementary Methods S4 | Functional assays (uptake, macrophage studies) |
| Supplementary Methods S5 | Proteomic profiling |
| Supplementary Methods S6  Supplementary Methods S7 | Clinical specimen collection  Bulk RNA-seq sequencing and bioinformatic analysis |
| Supplementary Figures S1–S9 | Multi-omics, EV validation, in vivo outcomes |
| Table S1 | qPCR primer sequences |

S1. Major Reagents and Instruments

Key reagents included: Q-VD-Oph (apoptosis inhibitor), DFO (lysosomal membrane permeabilization inhibitor), Hsp70, NAC (antioxidant), Nec-1s (necroptosis inhibitor), and G-CSF (granulocyte colony-stimulating factor), all comprising the CLON-G compound treatment; Lipopolysaccharide (LPS, Solarbio, L8880); Fetal Bovine Serum (Shanghai Shuangru Biological Technology Co., Ltd.); RPMI 1640 medium (Suzhou Junxin Biotechnology Co., Ltd.); PE-Ly6G and CD55 antibodies for flow cytometry; Gelatin type A (Bloom strength 300) was purchased from Sigma-Aldrich (St. Louis, MO, USA). Microbial transglutaminase (mTG) was obtained from Shanghai Yuanye Bio-Technology Co., Ltd.

Primary instruments comprised: a CO₂ incubator (Thermo Fisher Scientific), a low-speed centrifuge (Shanghai Fengchi), an upright optical microscope (Nikon), a flow cytometer (BD FACS Canto II), a nanoparticle tracking analyzer (ZetaView PMX 110), a transmission electron microscope (Hitachi H-7650), and a fluorescence inverted microscope (Keyence BZX810, Osaka, Japan).

For bulk RNA-seq, RNA extraction and library preparation were performed using the Magnetic Bead Animal Tissue Total RNA Extraction Kit (Servicebio, G3690), Hieff NGS mRNA Isolation Master Kit V2 (Yeasen, 12629ES96), and Hieff NGS EvoMax RNA Library Prep Kit (dNTP) (Yeasen, 12341ES97). Sequencing was performed using the DNBSEQ-T7RS platform with FCL PE150 V3.0 reagents (MGI, 940-000266-00).

S2. Isolation of Bone Marrow Neutrophils

Following established protocols (Fan et al., 2021) with slight modifications, neutrophils were isolated from bone marrow of 8-week-old male C57BL/6 mice. Briefly, femurs and tibias were dissected and flushed with 5 mL of HBSS buffer (Gibco, Cat# 14175-095) containing 15 mM EDTA (Sigma-Aldrich, Cat# E9884) and 0.5% BSA (Sigma-Aldrich, Cat# A9647). The collected cell suspension was centrifuged at 400 × g for 5 min at 4 °C. The pellet was resuspended in 3 mL HBSS and carefully layered onto a discontinuous Percoll gradient (52%, 62%, 76% in HBSS; Cytiva, Cat# 17089101) followed by centrifugation at 1,060 × g for 30 min at 4 °C without brake. Cells at the 62%–76% interface were carefully aspirated, washed with HBSS, and residual red blood cells were removed using Histopaque-1119 (Sigma-Aldrich, Cat# 11191) density centrifugation according to the manufacturer's instructions. Viable cells were counted using trypan blue exclusion. This procedure yielded neutrophils with a purity of >90% (confirmed by flow cytometry using PE-conjugated anti-Ly6G antibody, BD Biosciences, Cat# 551461) and an approximate yield of 1 × 10⁷ cells per mouse. Purity assessment was performed immediately after isolation and prior to experimental use.

Neutrophil Culture Conditions

To prolong neutrophil survival and inhibit multiple cell death pathways in vitro, neutrophils were cultured according to previously reported methods (Hsu AY et al., as cited in the main text). Cells were cultured in RPMI 1640 basal medium (Suzhou Junxin Biotechnology Co., Ltd., Cat# JXM0301) supplemented with 20% fetal bovine serum (FBS; Shanghai Shuangru Biological Technology Co., Ltd.), 100 U/mL penicillin, 100 μg/mL streptomycin, and the CLON-G compound mixture containing Q-VD-Oph (50 μM), deferoxamine mesylate (DFO, 1 μM), recombinant human Hsp70 protein (10 pM), N-acetylcysteine (NAC, 1 mM), Necrostatin-1s (Nec-1s, 10 μM), and recombinant mouse G-CSF (10 ng/mL). Neutrophils were seeded at 2 × 10⁶ cells/mL in 24-well plates and assigned to four experimental groups: (1) Control (CLON-G only), (2) high glucose (HG, 35 mM D-glucose), (3) lipopolysaccharide (LPS, 100 ng/mL; Solarbio, Cat# L8880), and (4) HG + LPS. An osmotic control was prepared by adding 19.5 mM L-glucose to the control medium. Cells were incubated at 37 °C with 5% CO₂ for 24 or 48 h before collection for downstream flow-cytometric analysis or NEV isolation.

Flow Cytometric Analysis

To assess isolation purity and CD55 expression under different conditions, neutrophils were harvested, washed twice with cold PBS containing 2% FBS, and resuspended in FACS buffer. Fc receptors were blocked with anti-mouse CD16/CD32 antibody (BD Biosciences, Cat# 553141, 1:100) for 15 min at 4 °C. Cells were then stained with PE-conjugated anti-mouse Ly6G antibody (BD Biosciences, Cat# 551461, 1:50) and an anti-mouse CD55 antibody, with CD55-associated fluorescence detected in the green fluorescence channel. After staining, cells were washed and analyzed by flow cytometry. Neutrophils were identified based on Ly6G expression and viability gating, and CD55 expression was quantified within the neutrophil population.

RT-qPCR Validation of the Atlas-Derived C2-Associated Neutrophil Program

To directly examine the molecular correspondence between HG+LPS-conditioned neutrophils and the atlas-defined C2 population, candidate genes were selected from the neutrophil-state signatures identified by scRNA-seq. The gene panel was designed to include both C2-associated positive markers and non-C2 state comparators rather than a nonspecific inflammatory gene set.

Specifically, *Cd55, Ltf, Chil3,* and *Camp* were selected as representative C2-associated genes. *Camp, Chil3,* and *Ltf* were among the characteristic genes enriched in the C2 cluster, whereas *Cd55* was preferentially expressed within C2 and was additionally selected because its encoded surface protein provides the complement-regulatory function central to the subsequent aNEV engineering strategy. To assess whether HG+LPS conditioning preferentially reproduced C2-associated characteristics rather than broadly activating genes from multiple neutrophil states, *Csf3* and *Jun* were included as representative comparator genes associated predominantly with the C0 and C1 populations, respectively.

Freshly isolated murine bone marrow neutrophils were seeded at 2 × 10⁶ cells mL⁻¹ and cultured for 24 h under four conditions: NT, HG (35 mM D-glucose), LPS (100 ng mL⁻¹), or combined HG+LPS stimulation. Following conditioning, cells were harvested and total RNA was isolated. RNA was reverse-transcribed into cDNA and subjected to quantitative real-time PCR using gene-specific primers for *Cd55, Ltf, Chil3, Camp, Csf3,* and *Jun*. For RT-qPCR validation of C2-associated genes under normal- and high-glucose conditions, mouse ***Hprt1*** was used as the endogenous reference gene instead of *Gapdh* to minimize potential normalization bias associated with glucose-dependent changes in glycolytic gene expression. *R*elative mRNA expression was calculated using the 2^-ΔΔCt^ method. Primer sequences are provided in Supplementary Table S1. Biological replicates were analyzed independently, and data are presented as mean ± SD.

S3. Extraction and Characterization of NEVs

Cell culture supernatants were first centrifuged at 1,500 × g for 5 min to remove cells. The collected supernatant was then centrifuged at 3,000 × g for 20 min at 4 °C to obtain the NEV-enriched pellet. The pellet was washed once with PBS, resuspended in 500 μL sterile PBS, and stored at −80 °C or immediately used for downstream analyses.

Nanoparticle Tracking Analysis (NTA)

Particle size distribution and concentration were determined using a ZetaView PMX 110 nanoparticle tracking analyzer (Particle Metrix) equipped with a 488 nm laser and CMOS camera. Samples were appropriately diluted in PBS (typically 1:1,000–1:10,000) to achieve 20–100 particles per frame. Measurements were performed at 11 positions per sample with three cycles of reading at each position. Camera sensitivity was set to 80%, and shutter speed to 100. Data were analyzed using ZetaView software (version 8.04.02) with automatic threshold and minimum area settings.

Transmission Electron Microscopy (TEM)

For transmission electron microscopy (TEM) analysis of neutrophil-derived extracellular vesicles (NEVs), 10 μL of NEV suspension was adjusted to an appropriate concentration and applied onto carbon-coated copper grids (400 mesh, Zhongjingkeyi Technology, Cat# BZ11032b). The sample was allowed to adsorb for 1 min at room temperature, and excess liquid was removed with filter paper. Negative staining was performed by applying 10 μL of 2% uranyl acetate (Shanghai Acmec Biochemical Technology, Cat# U25690-1g) onto the grid for 10 s, followed by blotting with filter paper to remove excess stain. The grids were air-dried for several minutes at room temperature. Imaging was performed using a transmission electron microscope (H-7650, Hitachi, Tokyo, Japan) operated at 100 kV. Images were captured at magnifications of ×15,000 (wide-field overview) and ×60,000 (close-up detail) to visualize both general distribution and ultrastructural morphology. Representative images from three independent NEV preparations were acquired. Vesicle diameter was measured using ImageJ software (version 1.54, NIH) from at least 100 individual particles per sample. Only circular or cup-shaped structures with distinct lipid bilayers and diameters within the range of 50–200 nm were counted as EVs. For each experimental condition, the mean diameter, mode diameter, and size distribution were calculated and reported as mean ± standard deviation.

Uranyl acetate was handled according to institutional radiation safety protocols. Waste was collected in designated containers for proper disposal.

Western Blot Analysis

For Western blot analysis, NEV samples and corresponding neutrophil samples were lysed in RIPA lysis buffer supplemented with protease inhibitor. Samples were lysed for 30 min and centrifuged at 12,000 rpm at 4 °C for 5 min. The supernatants were collected, and protein concentrations were determined using a BCA protein assay. Based on the measured protein concentrations, appropriate amounts of protein were mixed with 6× loading buffer and heated in a boiling water bath for 10 min before loading onto SDS-PAGE gels.

Electrophoresis was performed using a standard polyacrylamide gel system. After sample loading, proteins were initially separated through the stacking gel at 90 V for 30 min, followed by electrophoresis through the separating gel at 120 V for 30 min. Electrophoresis was terminated when the bromophenol blue dye front reached the bottom of the gel.

Proteins were transferred to PVDF membranes using a wet-transfer system. Before transfer, PVDF membranes were immersed in methanol for 1–2 min and subsequently equilibrated in ice-cold transfer buffer for 5 min. The gel was equilibrated in the same ice-cold transfer buffer for 3–5 min. Transfer buffer contained 25 mM Tris, 192 mM glycine, and 20% ethanol. The transfer sandwich was assembled with the gel facing the cathode and the PVDF membrane facing the anode, and protein transfer was performed at a constant current of 230 mA for 120 min.

Following transfer, membranes were physically sectioned according to the expected molecular-weight ranges of the target proteins before primary-antibody incubation, while retaining the corresponding molecular-weight marker lane for orientation. Individual membrane sections were subsequently blocked with 10% non-fat milk in TBST for 1 h at room temperature.

Membranes were incubated overnight at 4 °C with primary antibodies against CD55, TSG101, Alix, CD81, Ly6G, and β-Actin. Antibody information available from the experimental records was as follows: rabbit anti-CD55 (1:1,000, Proteintech, Cat# 26580-1-AP), rabbit anti-TSG101 (1:1,000, Abcam, Cat# ab125011), rabbit anti-Alix (1:1,000, Abcam, Cat# ab186429), rabbit anti-CD81 (1:1,000, Abcam, Cat# ab109201), and mouse anti-β-Actin (1:4,000, ABways, Cat# ab0035). The specific vendor and catalog information for the Ly6G antibody were not retained in the available experimental records.

After primary-antibody incubation, membranes were washed three times with TBST for 10 min each and subsequently incubated with the appropriate HRP-conjugated secondary antibody for 1 h at room temperature. Membranes were then washed three times with TBST for 10 min each.

For chemiluminescent detection, equal volumes of ECL reagents A and B were mixed and applied to the protein side of the membrane. After incubation in the dark for 5 min, excess reagent was carefully removed, and chemiluminescent signals were recorded using an imaging system.

Nano-Flow Cytometry (NanoFCM)

CD55 surface expression on neutrophil-derived extracellular vesicles (NEVs) was quantified using a nano-flow cytometer (Flow NanoAnalyzer, NanoFCM Bioscience, Xiamen, China; instrument serial number: FNAU30E25022122) equipped with dual lasers (488 nm blue laser at 10/50 mW power setting and 638 nm red laser at 10/100 mW power setting) and a photomultiplier tube detector array. NEV samples were diluted in sterile PBS to achieve appropriate particle concentrations for analysis. For CD55 detection, NEVs were incubated with FITC-conjugated anti-mouse CD55 antibody (1:50 dilution, BioLegend, Cat# 131807, clone RIKO-3) or FITC-conjugated isotype-matched IgG2bκ control antibody (1:50 dilution, BioLegend, Cat# 400607, clone MPC-11) for 30 min at room temperature in the dark. After washing twice with PBS (10,000 × g, 30 min, 4 °C) to remove unbound antibody, samples were resuspended in sterile PBS for analysis. Fluorescent silica nanosphere standards (100–500 nm diameter, NanoFCM Bioscience) were used for side scatter (SS-A) calibration and to establish the fluorescence intensity baseline. Samples were analyzed at a sample pressure of 1.0 kPa, corresponding to a sample flow rate of approximately 27.87 nL/min. Data acquisition was performed using NF Profession software (version 3.0, NanoFCM Bioscience) with the following instrument settings: side scatter decay (SS Decay) at 0.2%, threshold/subtraction parameters set at 21.86, 14.71, and 26.82, and minimum width (Min Width) at 0.3 ms. For each sample, particle events were recorded for 1–2 min until sufficient events (>2,000 particles) were captured. The blank control (PBS only) consistently yielded <10 events per acquisition, confirming minimal background contamination. CD55 positivity was determined by applying a fluorescence intensity threshold gate based on the 99.5th percentile of the isotype control-stained NEV population, with particles exceeding this threshold classified as CD55-positive. The net CD55 positivity rate was calculated by subtracting the isotype control background (typically <0.5%) from the anti-CD55 staining percentage. For quantitative comparison across stimulation conditions, NEV particle concentrations were normalized to the initial neutrophil cell count and reported as particles per cell. All samples were analyzed in technical triplicate, with at least three independent biological replicates per experimental condition.

S4. Macrophage Uptake Assay

EVs were first incubated with a PE-conjugated anti-CD55 antibody at 37 °C for 1 h and subsequently labeled with the green fluorescent membrane dye PKH67 (Sigma-Aldrich, MINI67). Briefly, EVs were resuspended in 0.5 mL Diluent C, while 4 μL PKH67 was separately diluted in 0.5 mL Diluent C. The two solutions were mixed and incubated for 4 min at room temperature in the dark. The labeling reaction was quenched with 5% BSA, followed by ultracentrifugation at 4 °C to remove residual dye. The labeled EV pellet was resuspended in 100 μL PBS and stored at 4 °C in the dark until use. Labeled EVs were then added to recipient macrophages and incubated at 37 °C for 8 h. Cells were washed three times with PBS, fixed with 4% paraformaldehyde for 10 min, permeabilized with 0.5% Triton X-100 for 5 min at 25 °C, and counterstained with DAPI (Solarbio, C0065). After washing, EV uptake was examined using a Nikon CSU-W1 SoRa confocal microscope.

S5. Proteomic Profiling of NEV

For proteomic analysis, neutrophil-derived extracellular vesicles (NEVs) were isolated from control (CLON-G only) and HG+LPS-stimulated neutrophils (three independent biological replicates per group, designated C-1 through C-3 and HG-LPS-1 through HG-LPS-3, respectively). NEV pellets were lysed by sonication (1 s on /1 s off, cumulative 2 min) and centrifuged at 14,000 × g for 20 min at 4 °C. The supernatant was collected, and protein concentration was determined by BCA assay. For each sample, 25 μL aliquot was reserved for BCA quantification, with the remainder stored at −80 °C. Proteomic sample preparation was performed using an integrated spin column workflow (Echo Biotech, Beijing, China). Briefly, proteins were pre-concentrated on mixed SCX/SAX ion-exchange beads, subjected to in-bead reduction and alkylation, and digested with sequencing-grade trypsin. The resulting peptides were desalted on C1 reverse-phase membranes, eluted with high-salt buffer, and lyophilized to dryness. Dried peptides were resuspended in 10 μL mobile phase A (100% water, 0.1% formic acid), centrifuged at 14,000 × g for 20 min at 4 °C, and 200 ng peptide per sample was injected for LC-MS/MS analysis.

Liquid chromatography was performed on a Vanquish Neo UHPLC system (Thermo Fisher Scientific) equipped with a C18 trap column (5 mm × 300 μm, 5 μm particle size, Thermo Fisher Scientific, Cat# 174500) and an analytical column (PepMap Neo UHPLC, 150 μm × 15 cm, 2 μm particle size, Thermo Fisher Scientific, Cat# ES906). The column oven was maintained at 50 °C. Peptides were separated at a flow rate of 2.5 nL/min using the following gradient program: 4–5% mobile phase B (80% acetonitrile, 0.1% formic acid) from 0 to 0.2 min, 5–75% B from 0.2 to 4 min, 75–65% B from 4 to 5.8 min, column wash at 99% B from 5.8 to 6.2 min, 99% B from 6.2 to 6.9 min, and stop run at 6.9 min (mobile phase A: 100% water, 0.1% formic acid).

Mass spectrometric detection was performed on an Orbitrap Astral mass spectrometer (Thermo Fisher Scientific) equipped with a quadrupole mass filter, an Orbitrap analyzer for high-resolution full-scan MS, and an Astral analyzer for high-speed MS/MS acquisition. The ion source was operated in positive electrospray ionization (ESI) mode with an EASY-Spray source. Ion spray voltage was set at 1.9 kV, and ion transfer tube temperature at 290 °C. Data-independent acquisition was employed. Full MS scans covered m/z 380–980 at a resolution of 240,000 (at m/z 200), with an AGC target of 500%. The precursor isolation width was 2 Th with 300 DIA windows, while NCE was set to 25%. MS/MS spectra were acquired over m/z 150–2,000 using the Astral analyzer at a resolution of 80,000 and a maximum injection time of 3 ms.

Raw data files (.raw) were processed using DIA-NN software (version 1.8.1, University of California, San Francisco) against the Mus musculus UniProt database (release 20250611, 17,237 entries). Search parameters included: fragment ion quantification, trypsin as digestion enzyme with maximum 2 missed cleavages, carbamidomethylation of cysteine as fixed modification, and acetylation of protein N-terminus and oxidation of methionine as variable modifications. Protein quantification values were median-normalized across samples to correct for systematic experimental bias. Proteins detected in fewer than 50% of samples within either group were filtered out, and missing values were imputed using the k-nearest neighbors (KNN) algorithm. Differentially expressed proteins (DEPs) were identified between control and HG+LPS groups using Student's t-test, with significance thresholds set at fold change ≥ 1.5 or ≤ 1/1.5 and P-value ≤ 0.05. Functional annotation and enrichment analyses were performed using Gene Ontology (GO) and Kyoto Encyclopedia of Genes and Genomes (KEGG) databases, with hypergeometric distribution tests and multiple-testing correction (Benjamini-Hochberg FDR). Subcellular localization was annotated using LocTree3 (score > 40).

S6. Clinical Specimen Collection and Processing

**Ethical Approval and Patient Consent.** This retrospective study using archived clinical specimens was approved by the Ethics Committee of Shanghai Sixth People’s Hospital (Approval No: 2025-KY-195(K)) and conducted in accordance with the Declaration of Helsinki. Written informed consent for the research use of archived specimens and associated anonymized data and images was obtained from all included participants.

**Patient Selection and Eligibility Criteria.** Eligible cases were retrospectively identified from patients with type 2 diabetes mellitus (T2DM) who had undergone surgical treatment for diabetic foot ulcers (DFU). Inclusion criteria were: (i) age 18–70 years; (ii) clinically diagnosed DFU requiring debridement or amputation; and (iii) written informed consent. Exclusion criteria included severe systemic diseases, malignancy, AIDS, psychiatric disorders, or other conditions considered unsuitable for inclusion.

**Tissue Sampling and Processing.** Archived full-thickness dorsal foot skin specimens (~1.0 cm × 1.0 cm × 0.3 cm) obtained during DFU surgery were used. Specimens were fixed in 4% paraformaldehyde, dehydrated through graded ethanol, cleared in xylene, embedded in paraffin, and sectioned at 4 μm. Sections were used for H&E staining, Masson’s trichrome staining, and immunofluorescence analysis.

S7. Bulk RNA-seq Sequencing and Bioinformatic Analysis

Full-thickness wound tissues collected on postoperative day 7 (POD 7) from the Gel and Gel/aNEV groups were used for bulk RNA sequencing, with three independent biological replicates per group. Total RNA was extracted using the Magnetic Bead Animal Tissue Total RNA Extraction Kit (Servicebio, Cat. G3690). RNA purity was assessed using NanoDrop, RNA concentration was determined using the Equalbit RNA BR Assay Kit (Vazyme, Cat. EQ212-02), and RNA integrity was evaluated by agarose gel electrophoresis. Approximately 2 μg of total RNA was used for library preparation. Poly(A)+ mRNA was isolated using the Hieff NGS mRNA Isolation Master Kit V2 (Yeasen, Cat. 12629ES96), and non-strand-specific mRNA libraries were constructed using the Hieff NGS EvoMax RNA Library Prep Kit (dNTP) (Yeasen, Cat. 12341ES97). Libraries were quantified using the Equalbit 1× dsDNA HS Assay Kit (Vazyme, Cat. EQ121-02), circularized using the Hieff NGS Dual Barcode Fast-Pace DNA Cyclization Kit for MGI (Yeasen, Cat. 13340ES96), and quantified after circularization using the Equalbit ssDNA Assay Kit (Vazyme, Cat. EQ311-02). Sequencing was performed on a DNBSEQ-T7RS system using FCL PE150 V3.0 reagents (MGI, Cat. 940-000266-00), generating paired-end 150-bp reads.

Raw FASTQ reads were processed using fastp v0.23.2 for adapter removal and quality filtering. Clean paired-end reads were aligned to the *Mus musculus* GRCm39 reference genome with M31 annotation (Ensembl release 108) using HISAT2 v2.2.1. Gene-level expression was quantified using StringTie v2.2.1, and gene-level read counts, transcripts per million (TPM), and fragments per kilobase of transcript per million mapped reads (FPKM) were generated. Differential expression between the Gel/aNEV and Gel groups was analyzed using DESeq2 v1.42.1 with Benjamini–Hochberg correction. Genes with |log_2_(fold change)| > 1 and adjusted *P* < 0.05 were considered differentially expressed. Gene Ontology (GO), Kyoto Encyclopedia of Genes and Genomes (KEGG), and Gene Set Enrichment Analysis (GSEA) were performed using clusterProfiler v4.10.1.

**Table S1. Quantitative Real-time PCR primer sequences.**

| Gene | Forward | Reverse |
| --- | --- | --- |
| *Il1b* | TTCAAATCTCGCAGCAGCACATC | CGTCACACACCAGCAGGTTATCAT |
| *Il4* | GGTCTCAACCCCCAGCTAGT | GCCGATGATCTCTCTCAAGTGAT |
| *Il6* | CTGCAAGAGACTTCCATCCAG | AGTGGTATAGACAGGTCTGTTGG |
| *Il10* | GTTGCCAAGCCTTATCGGAAATG | TCACTCTTCACCTGCTCCACTGC |
| *Ccr7* | CCCAGGGAAACCCAGGAAAAAC | ACCGTGGTATTCTCGCCGATGTAG |
| *Mmp9* | GCTGGCAGAGGCATACTTGTAC | CAGTATGTGATGTTATGATGGTCCC |
| *C3ar1* | CACAGCCATTCCTAACATCTCC | GCGTCAGCGTTCAGTGTATTG |
| *C5ar1* | GCAGCCCTTATCATCTACTCGG | CCGCCAGATTCAGAAACCAG |
| *C5ar2* | TGGTGTTCATGGCCGGTTG | CCTGGAGTGACTTGCAGAGTTGTT |
| *Nlrp3* | TAAGAACTGTCATAGGGTCAAAACG | GTCTGGAAGAACAGGCAACATG |
| *Lrp1* | GCCTGCCAACTGTACCCAAAT | TATCTCCGCAGTCGTTGTCGT |
| *Stat6* | CTCTGCCCTTGGTGGTCATC | CTCGCTCAGCCACCACAAA |
| *Pparg* | CAGAAGTGCCTTGCTGTGGG | TGGTCAGCGGGAAGGACTTTAT |
| *Cfh* | CGCCCTGGATACCGAACACT | CTGCCAGCCTAAAGGACCCA |
| *Itgam* | CTGTCCCTGGCTGTTTCTACTG | GGAGCCATCAATCAAGAAGACAA |
| *Camp* | TGAAGGAGACTGTATGTGGCAAG | CTTGAACCGAAAGGGCTGTG |
| *Chil3* | CAAGGCTGCTACTCACTTCCAC | GCTGGAAATCCCACAATGAGC |
| *Ltf* | GCCAACAAATGTGCCTCTTCC | TACTGGTCCCTTTCGGCTTTATT |
| *Csf3* | CCCACCTTGGACTTGCTTCA | CGCTGGAAGGCAGAAGTGAA |
| *Jun* | CCTTCTACGACGATGCCCTC | GGGTCGGTGTAGTGGTGATGT |
| *Cd55* | CAGAAAGACTGAGTTTTGCATCC | GAGCAACTGGAGACCATACTAAATC |
| *Hprt1* | AGCCAAATACAAAGCCTAA | AGGACTAGAACACCTGCTAAT |
| *Gapdh* | CGGTGCTGAGTATGTCGTGGAGTC | GGCGGAGATGATGACCCTTTTG |

Primers were synthesized by Servicebio (Wuhan, China) or BioTNT Co., Ltd. (Shanghai, China).

**Figure S1. Trajectory inference and pseudotime-dependent gene expression dynamics of neutrophil subsets in diabetic wounds.** (A) Differentiation trajectory of neutrophils constructed using Monocle 2 algorithm, with left panel colored by cell subsets (C0, C1, C2) and right panel colored by pseudotime gradient, illustrating the differentiation path from C0 through C1 toward C2 state. (B) Expression dynamics of the top 6 differentially expressed genes along the pseudotime axis, including bar plots showing expression levels across subsets (left), violin plots displaying expression distribution (middle), and scatter plots demonstrating expression trends over pseudotime (right). (C) Heatmap clustering of top 100 pseudotime-dependent differentially expressed genes (left) and Gene Ontology Biological Process (GO-BP) enrichment analysis of subset-specific genes (right); the heatmap reveals three major gene clusters corresponding to distinct expression patterns at different differentiation stages. (D) Stacked bar chart showing the proportional distribution of neutrophil subsets (C0, C1, C2) across differentiation states (State 1-7). (E) Density distribution curves of each neutrophil subset along the pseudotime axis, indicating that C0 subset predominantly occupies the early pseudotime while C2 subset concentrates at the terminal stage, suggesting distinct temporal differentiation characteristics.

**Figure S2. Differential cell-cell communication networks between normal and diabetic wound healing and neutrophil subset interaction analysis.** (A) Statistical comparison of significantly altered cell-cell communications between normal mouse wounds (Normal wound) and diabetic mouse wounds (Diabetic wound). Left panel shows the number of differential interactions (red bars: decreased communications; cyan bars: increased communications), and right panel shows differential interaction strength (red bars: reduced strength; cyan bars: enhanced strength). Both the number and strength of cellular communications were significantly elevated in diabetic wounds compared to normal wounds, indicating aberrantly activated inflammatory intercellular communication in diabetic wounds. (B) Overall cell-cell interaction heatmaps based on CellChat algorithm, with left panel displaying differential number of interactions and right panel showing differential interaction strength between cell types. Rows represent sender cells and columns represent receiver cells; color intensity indicates relative difference values, with red denoting enhanced/increased interactions and blue indicating weakened/reduced interactions in diabetic wounds; bar plots on the right show the overall differential contribution of each cell type. (C) Ligand-receptor communication network dot plot showing neutrophil subsets (C0, C1, C2) as signal senders to other cell types (endothelial cells, fibroblasts, macrophages, etc.). Dot size represents communication probability and color indicates statistical significance (P value), revealing that C2 subset exhibits specific communication advantages toward fibroblasts and macrophages. (D) Reverse communication network dot plot showing other cell types as signal senders to neutrophil subsets, uncovering significant paracrine regulatory loops between macrophages and neutrophils, which were notably enhanced in diabetic wounds.

**Figure S3. Histological characteristics and infiltration pattern of CD55⁺ neutrophils during early wound healing in normal mouse skin.** H&E staining of wounds on days 1 and 3 showing the gradual resolution of inflammation and epithelial regeneration. Immunofluorescence dual staining of LY6G and CD55 demonstrating massive infiltration of CD55⁺ neutrophils at D1 with significant reduction at D3.

**Figure S4.** **Phenotypic and molecular validation of the HG+LPS-conditioned C2-like neutrophil state.** **(A)** Representative flow cytometry histograms showing Ly6G⁺ neutrophil purity following Percoll gradient isolation. **(B)** Representative dot plots of CD55 and Ly6G co-expression under indicated stimulation conditions at 24 h and 48 h. **(C)** Quantification of neutrophil purity (n = 3). **(D)** Quantitative analysis of CD55⁺/Ly6G⁺ double-positive neutrophil percentages (n = 3). **(E)** RT-qPCR analysis of C2-associated genes (*Cd55, Ltf, Chil3,* and *Camp*) together with the C0-associated gene *Csf3* and the C1-associated gene *Jun* after 24 h of NT, HG, LPS, or HG+LPS treatment. HG+LPS conditioning prominently increased *Ltf, Chil3,* and *Camp* expression and maintained elevated *Cd55* expression relative to untreated neutrophils, whereas *Csf3* showed no significant induction. *Jun* was also increased under HG+LPS stimulation, indicating that the induced state recapitulates selected C2-associated features rather than constituting an exact transcriptional replica of the endogenous C2 population. Gene expression was normalized to the mouse reference gene *Hprt1*. (n = 3). * *P* < 0.05, ***P* < 0.01, ****P* < 0.001; n.s., not significant.

**Figure S5. Macrophage internalization of neutrophil-derived extracellular vesicles (NEVs).** (A) Merged image showing PKH67-positive NEVs (green) internalized by RAW 264.7 macrophages with DAPI-stained nuclei (blue). (B) FITC channel detecting PKH67 fluorescence signal. (C) DAPI channel showing nuclear counterstaining. (D) PE channel demonstrating absence of detectable PE-anti-CD55 signal following NEV internalization. (E) Bright-field (BF) image revealing macrophage morphology and cell boundaries.

**Figure S6. Verification of aNEV incorporation within the hydrogel and nanoparticle persistence after 14-day incubation in diabetic wound-mimicking fluid.** **(A)** Representative fluorescence images showing PKH67-associated signals in the hydrogel. Gel incubated with PKH67 alone was used as the dye-background control, whereas Gel/aNEV contained PKH67-labeled aNEVs to visualize vesicle incorporation within the hydrogel matrix. Green fluorescence indicates PKH67-associated signals. **(B)** Representative nanoparticle tracking analysis (NTA) size-distribution profiles of residual materials recovered from Gel and Gel/aNEV after 14 days of incubation in the defined diabetic wound-mimicking simulated wound fluid (DM-SWF) at 37 °C. NTA of the residual Gel/aNEV sample revealed an aNEV-sized nanoparticle population after 14 days of incubation, whereas the residual blank Gel sample predominantly contained smaller particles.

**Figure S7. GSEA of inflammatory, complement-related, and tissue-repair pathways in Gel/aNEV-treated wounds.** **(A)** GSEA plot of cytokine–cytokine receptor interaction showing negative enrichment in the Gel/aNEV group. **(B)** GSEA plot of chemokine signaling pathway showing negative enrichment. **(C)** GSEA plot of Wnt signaling pathway showing positive enrichment, consistent with activation of tissue-repair-associated signaling. **(D)** GSEA plot of complement and coagulation cascades showing negative enrichment. NES, nominal *P* values, and adjusted *P* values are indicated within each enrichment plot. **(E)** Summary of therapeutically relevant GO- and KEGG-based GSEA terms associated with inflammation, complement regulation, tissue repair, and angiogenesis. Negative NES values indicate enrichment toward the Gel group, whereas positive NES values indicate enrichment toward the Gel/aNEV group. GO-GSEA showed negative enrichment of immune- and complement-associated programs, including leukocyte-mediated immunity, activation of innate immune response, and complement activation pathways, together with positive enrichment of epidermis development, keratinocyte differentiation, epidermal cell differentiation, endothelial development, and epidermal wound-healing programs. KEGG-GSEA similarly revealed negative enrichment of cytokine/chemokine-associated inflammatory pathways and complement/coagulation cascades, whereas Wnt, TGF-β, VEGF, and cAMP signaling showed positive enrichment trends. Bar length represents the normalized enrichment score (NES), and the corresponding FDR value is indicated for each pathway. Visualization code for panel E was generated and refined with assistance from OpenAI Codex using the GPT-5.6 Sol model. The plot was generated from the authors’ underlying data and statistical results and was reviewed and verified by the authors.

**Figure S8. Integrated transcriptomic–proteomic enrichment analysis reveals coordinated oxidative stress, inflammatory, and tissue-repair pathways. (A)** Gene Ontology (GO) enrichment analysis of the integrated transcriptomic–proteomic dataset, displayed across biological process (BP), cellular component (CC), and molecular function (MF) categories. Enriched BP terms included responses to reactive oxygen species, oxidative stress, hypoxia, and epithelial-cell proliferation; CC terms included membrane- and extracellular-matrix-associated components; and MF terms included antioxidant activity, peroxidase/peroxiredoxin activity, integrin binding, and growth factor receptor-associated functions. Bubble size represents the number of mapped molecules, color indicates −log10(*P* value), and the x-axis denotes the rich factor. **(B)** KEGG pathway enrichment analysis of the integrated dataset. Enriched pathways included TNF, PI3K–Akt, IL-17, focal adhesion, AGE–RAGE signaling in diabetic complications, lipid and atherosclerosis, and fluid shear stress and atherosclerosis, among others. Bar length represents −log10(*P* value), and color intensity indicates the number of mapped molecules.

**Figure S9. Mapping of original Western blot membrane sections corresponding to Figure 3E.**
